# Ablation of CCL17‐positive hippocampal neurons induces inflammation‐dependent epilepsy

**DOI:** 10.1111/epi.18200

**Published:** 2024-11-28

**Authors:** Judith Eberhard, Lukas Henning, Lorenz Fülle, Konrad Knöpper, Jana Böhringer, Frederike J. Graelmann, Lea Hänschke, Julia Kenzler, Frederic Brosseron, Michael T. Heneka, Ana I. Domingos, Stefanie Eyerich, Matthias Lochner, Heike Weighardt, Peter Bedner, Christian Steinhäuser, Irmgard Förster

**Affiliations:** ^1^ Immunology & Environment, Life and Medical Sciences Institute University of Bonn Bonn Germany; ^2^ Institute of Cellular Neurosciences, Medical Faculty University of Bonn Bonn Germany; ^3^ Deutsche Forschungsgemeinschaft Bonn Germany; ^4^ Business Development Europe Research Services, WuXi Biologics, Leverkusen, Germany; ^5^ Howard Hughes Medical Institute and Department of Microbiology and Immunology University of California, San Francisco San Francisco California USA; ^6^ Molecular Developmental Biology, Life and Medical Sciences Institute University of Bonn Bonn Germany; ^7^ Sanofi‐Aventis Deutschland Medical Operations General Medicines in Germany, Switzerland, Austria (GSA) Berlin Germany; ^8^ German Center for Neurodegenerative Diseases Bonn Germany; ^9^ Luxembourg Center for Systems Biomedicine University of Luxembourg Esch‐sur‐Alzette Luxembourg; ^10^ Department of Physiology, Anatomy, and Genetics University of Oxford Oxford UK; ^11^ Institute for Medical Microbiology, Immunology, and Hygiene Technical University of Munich Munich Germany; ^12^ ZAUM—Center of Allergy and Environment Technical University and Helmholtz Center Munich Munich Germany; ^13^ Institute of Medical Microbiology and Hospital Epidemiology Hannover Medical School Hannover Germany

**Keywords:** astrogliosis, diphtheria toxin, microgliosis, neurodegeneration, XPro1595

## Abstract

**Objective:**

Neuronal cell death and neuroinflammation are characteristic features of epilepsy, but it remains unclear whether neuronal cell death as such is causative for the development of epileptic seizures. To test this hypothesis, we established a novel mouse line permitting inducible ablation of pyramidal neurons by inserting simian diphtheria toxin (DT) receptor (DTR) cDNA into the *Ccl17* locus. The chemokine CCL17 is expressed in pyramidal CA1 neurons in adult mice controlling microglial quiescence.

**Methods:**

Seizure activity in CCL17‐DTR mice was analyzed by electroencephalographic recordings following treatment with DT for 3 consecutive days. Neuroinflammation and neuronal cell death were evaluated by (immuno)histochemistry. Pharmacological inhibition of TNFR1 signaling was achieved by treatment with XPro1595, a dominant‐negative inhibitor of soluble tumor necrosis factor.

**Results:**

Neuronal cell death was detectable 7 days (d7) after the first DT injection in heterozygous CCL17‐DTR mice. Spontaneous epileptic seizures were observed in the vast majority of mice, often with an initial peak at d6–9, followed by a period of reduced activity and a gradual increase during the 1‐month observation period. Microglial reactivity was overt from d5 after DT administration not only in the CA1 region but also in the CA2/CA3 area, shortly followed by astrogliosis. Reactive microgliosis and astrogliosis persisted until d30 and, together with neuronal loss and stratum radiatum shrinkage, reflected important features of human hippocampal sclerosis. Granule cell dispersion was detectable only 3 months after DT treatment. Application of XPro1595 significantly reduced chronic seizure burden without affecting the development of hippocampal sclerosis.

**Significance:**

In conclusion, our data demonstrate that sterile pyramidal neuronal death is sufficient to cause epilepsy in the absence of other pathological processes. The CCL17‐DTR mouse line may thus be a valuable model for further mechanistic studies on epilepsy and assessment of antiseizure medication.

## Graphical abstract

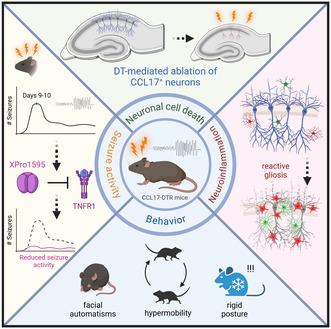

Ablation of CCL17‐expressing hippocampal neurons induces spontaneous epileptic seizures accompanied by neurodegeneration, gliosis and behavioral changes. Inhibition of soluble TNF by XPro1595 application reduces seizure burden without affecting neuroinflammation or neuronal cell loss.

**Key points**

- Inducible ablation of CCL17‐expressing cells models neuronal loss‐induced epilepsy.
- Neuronal cell death is sufficient to induce spontaneous recurrent seizures and neuroinflammation.
- Suppression of soluble TNF by XPro1595 reduces seizure burden but not neuroinflammation.

## 1 INTRODUCTION

Epilepsy is one of the most frequent neurological diseases, with a prevalence of 1% in the global population. It is defined by the International League Against Epilepsy as either the occurrence of at least two unprovoked seizures more than 24 h apart or a single unprovoked seizure followed by an enduring predisposition to have further seizures.^1^ In adults, the most common form of epilepsy is temporal lobe epilepsy (TLE), in which the hippocampus, amygdala, and parahippocampal gyrus are primarily affected. Currently, pharmacological treatment of epilepsy is effective in approximately two thirds of all epileptic patients,^2^ whereas the majority of TLE patients are nonresponders to antiseizure medication.^3^

It has been hypothesized that neuronal loss in response to an epileptogenic brain insult or trauma is sufficient to cause long‐lasting brain network disturbances that culminate in epilepsy.^4^ However, currently available and commonly employed models of chemoconvulsant‐ or electrical stimulation‐induced epilepsy, which are known to cause rapid neuronal death,^5^, ^6^, ^7^ induce a plethora of effects and thus lack specificity to examine this hypothesis. Therefore, it remains unclear whether neuronal loss is a cause or consequence of the epileptogenic process. Targeted ablation of neurons using genetic tools may represent a way to assess the potentially causal role of neuronal cell death in epileptogenesis.

Inducible expression of diphtheria toxin A (DTA)^8^ or expression of a diphtheria toxin receptor (DTR) under the control of a cell‐type specific promoter^9^ enables diphtheria toxin (DT)‐mediated targeted ablation of selective cell populations due to DT‐dependent blockade of protein synthesis. Cell‐type specific and inducible expression of DTA driven by the *CamKII* or *Nex1* promoter led to ablation of forebrain excitatory neurons^8^, ^10^ or pyramidal neurons in the hippocampus and cortex,^10^, ^11^ respectively. In both cases, mice developed cognitive impairment after induction of neuronal cell loss, accompanied by a long‐lasting neuroinflammatory response, but no information about the occurrence of epileptic seizures in these models is available.

In the current study, we present a novel mouse line with cell type‐specific expression of the DTR driven by the murine *Ccl17* (CC chemokine ligand 17) gene locus (CCL17‐DTR mice). CCL17 is a chemokine that has been originally found to be produced by dendritic cells and certain subsets of macrophages in the immune system.^12^, ^13^ Recently, we could demonstrate that CCL17 is also expressed in a subset of hippocampal CA1 pyramidal neurons in the adult brain, where it aids to maintain microglia in a quiescent, ramified state.^14^ Hence, expression of the DTR under control of the *Ccl17* promoter region would allow examination of the effects of selective pyramidal neuron ablation on brain function and epilepsy development. We demonstrate that transient treatment of heterozygous CCL17‐DTR mice (CCL17^DTR^ mice) with DT leads to a sterile and long‐lasting depletion of pyramidal neurons causing robust induction of persistent spontaneous recurrent seizures (SRSs) starting within a defined time period of 6 days (d6) after DT injection and is accompanied by local microglia reactivity and astrocytosis, eventually leading to hippocampal sclerosis (HS). We also demonstrate that the frequency of SRSs in CCL17^DTR^ mice, but neither neuronal cell loss nor neuroinflammation, was dependent on the presence of soluble tumor necrosis factor (sTNF) using a pharmacological inhibitor.

## 2 MATERIALS AND METHODS

A comprehensive description of the materials, methods, and analysis procedures is available in the Supporting Information provided with this article.

### 2.1 Animals

All experiments were performed in accordance with EU and local governmental regulations. Experiments were approved by the North Rhine–Westphalia State Agency for Nature, Environment, and Consumer Protection (84–02.04.2015.A393, 84–02.04.2016.A409, 81–02.04.2020.A420, 81–02.04.2021.A426).

### 2.2 Drugs

#### 2.2.1 Administration of DT, PEGylated DT, and phosphate‐buffered saline

**FIGURE 1:**
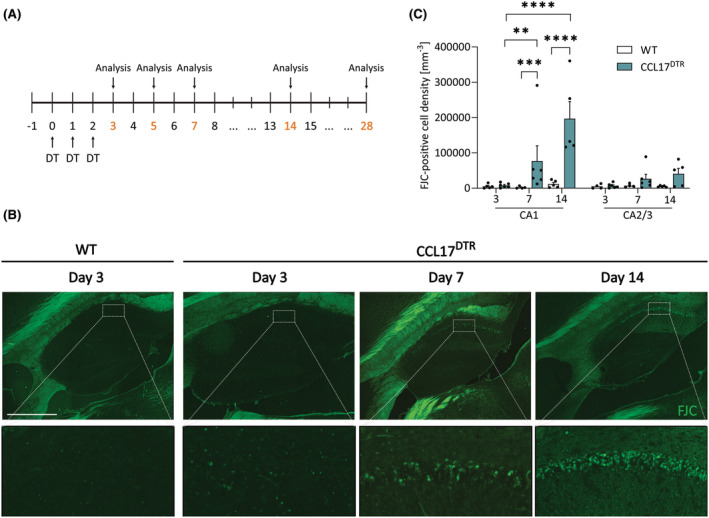
Diphtheria toxin (DT) induces neuronal degeneration in CCL17^DTR^ mice. (A) Timeline of experimental procedure. “DT” indicates intraperitoneal injection of .4 μg DT/mouse. End points of experiments are depicted in red. (B) CCL17^DTR^ mice and wild‐type (WT) mice received 150 μL phosphate‐buffered saline at day (d) −1 and .4 μg DT at d0, d1, and d2. Mice were perfused in situ, and brains were isolated at d3, d7, and d14. Forty‐micrometer brain sections were prepared and degenerating neurons detected by Fluoro Jade C labeling (FJC; green). Images were prepared using epifluorescence microscopy. Scale bar (500 μm) applies to upper panels. Lower panels depict magnifications of upper panel as indicated. Representative images are shown. (C) Density of FJC cells in the hippocampal CA1 and CA2/CA3 regions. Data were analyzed using a linear mixed‐model regression of Tukey‐transformed data (*λ* = .25). **p < .01, ***p < .001, ****p < .0001. *N* = 4–5 WT and *N* = 5–6 CCL17^DTR^ mice with *n* = 1 section/mouse.

Wild‐type (WT), CCL17^DTR^, and CCL17^DTR/DTR^ mice received intraperitoneal injections of .4 μg DT or .4 μg PEGylated DT (PEGyDT; PEGylation = covalent attachment of long unstructured polyethylene glycol chains^15^) dissolved in 150 μL phosphate‐buffered saline (PBS) on 3 consecutive days (d0, d1, and d2). In some experiments, WT mice and CCL17^DTR^ mice received 150 μL ip PBS 1 day before the first DT injection (d−1) as indicated in the legends to Figures 1 and 3.

#### 2.2.2 XPro1595 treatment

CCL17^DTR^ mice received three intraperitoneal injections of 10 mg/kg XPro1595 (10 mg/mL dissolved in .9% sterile sodium chloride) every 72 h starting 1 day before the first DT administration.

### 2.3 Electroencephalographic recordings and data analysis

Telemetric electroencephalographic (EEG) transmitters were implanted 5 days after the first DT administration at the House of Experimental Therapies, University Hospital Bonn, Germany. EEG recordings (24 h/day, 7 days/week) started immediately after transmitter implantation and continued until d30 after the start of DT treatment. EEG data were analyzed using NeuroScore (version 3.4.0) software (Data Sciences International) as described previously.^16^ A video surveillance system (Bascom) was used to monitor behavioral seizure activity.

### 2.4 Whole‐cell patch clamp recordings and biocytin‐loading of astrocytes

Mice were anesthetized with isoflurane (Piramal Healthcare) and decapitated. Coronal brain sections (200 μm) were prepared and transferred to artificial cerebrospinal fluid. To analyze gap junction (GJ) coupling, whole‐cell patch clamp recordings of Sulforhodamine 101‐positive astrocytes were performed during which astrocytes were filled with biocytin for 20 min at room temperature. Slices were subsequently used for immunohistochemical labeling of biocytin‐filled astrocytes to quantify astroglial coupling efficiency.

### 2.5 Immunohistochemistry and image analysis

Animals were anesthetized and transcardially perfused. Brains were removed, and coronal or parasagittal brain sections were prepared and utilized for (immuno)histochemical staining of microglia, astrocytes, neurons, and neurodegeneration. Imaging was performed using either confocal or epifluorescence microscopy. Analysis was performed semiautomatically using ImageJ (Fiji) or IMARIS 8.0 software. Parvalbumin (PV)‐positive neurons and biocytin‐positive cells were counted manually.

### 2.6 Statistical analysis

Statistical analyses and data visualization were performed either with GraphPad Prism 6 and 9, R,^17^ or OriginPro (OriginLab Corporation) software. Data are presented as mean value ± SEM or as boxplots representing median (line) and quartiles (25th and 75th percentile) with whiskers extending to the highest and lowest values within 1.5 times the interquartile range. The significance level was set at *p* < .05. The number of animals used per experiment is depicted as *N*. The number of sections analyzed per mouse is depicted as *n*.

## 3 RESULTS

### 3.1 Ablation of CCL17‐expressing neurons in CCL17^DTR^ mice

To examine the effects of selective pyramidal neuron depletion in the hippocampus on brain functioning and epilepsy development, we generated a mouse line expressing the simian DTR under control of the *Ccl17* promoter, which is expressed in a subset of hippocampal CA1 pyramidal neurons in mice.^14^ This approach enables DT‐mediated ablation of the DTR‐expressing cell population.^9^ First, we examined the extent and time course of pyramidal neuron depletion in CCL17^DTR^ mice subjected to three consecutive intraperitoneal injections of DT (.4 μg/mouse/day; Figure 1A). Three, 7, and 14 days following the first DT injection, mice were sacrificed and brains were harvested. Parasagittal sections were examined for neuronal degeneration by quantification of Fluoro‐Jade C (FJC)‐positive cells in the hippocampus (Figure 1B). WT mice exhibited no significant neuronal degeneration following DT administration at any time point analyzed (Figures 1B,C and S1A). In contrast, CCL17^DTR^ mice displayed FJC‐positive neurons in the hippocampal pyramidal layer at d7 and d14 after DT injection (Figure 1C). Besides a significant increase of degenerating neurons in the CA1 region, we also observed a few FJC‐positive cells in the CA2/CA3 region (Figure 1B,C). Importantly, injection of a blood–brain barrier (BBB)‐impermeable variant of DT, PEGyDT,^15^ did not cause hippocampal neurodegeneration in CCL17^DTR^ mice at d28, indicating that the cell death‐inducing effect of DT is due to direct action on hippocampal neurons rather than peripheral effects of DT (Figure S2A,C). Moreover, the extent of neurodegeneration was similar in CCL17^DTR^ versus CCL17^DTR/DTR^ mice 4 weeks after the start of DT administration, demonstrating that maximal DT‐induced cell death can already be achieved with heterozygous DTR expression (Figure S2B,C).

In conclusion, expression of a DTR under control of the *Ccl17* promoter constitutes a highly effective method to induce DT‐mediated neurodegeneration in the pyramidal layer of the hippocampus within a 2‐week time frame.

### 3.2 DT‐mediated cell death of CCL17‐positive pyramidal neurons induces epilepsy

**FIGURE 2:**
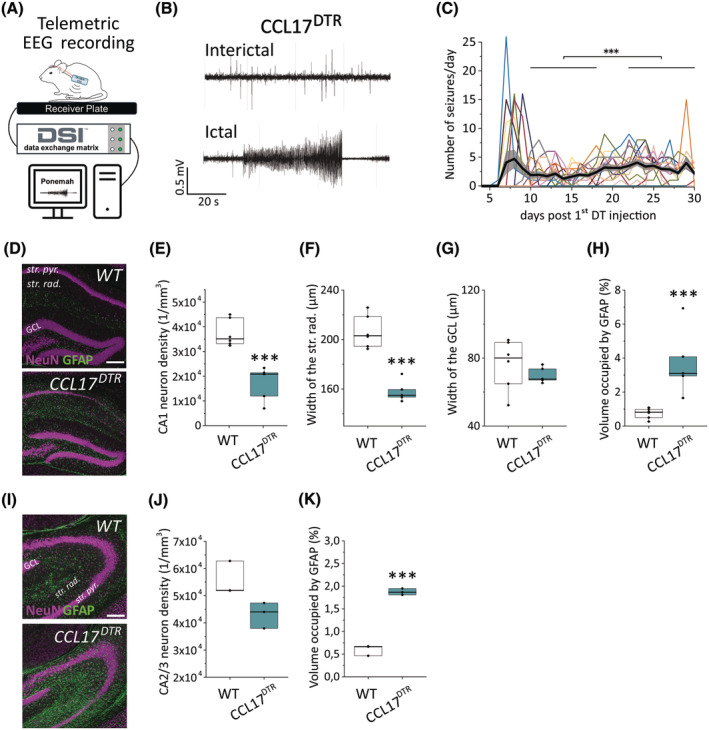
Diphtheria toxin (DT)‐mediated ablation of hippocampal CCL17‐positive neurons induces epileptiform activity and hippocampal damage. (A) Electrographic seizures were continuously detected via cortical electrodes (placed ~1 mm deep into the cortex, 3 mm posterior to bregma, and 1.5 mm lateral to midline) and telemetric transmitters. (B) Representative electroencephalographic (EEG) traces depicting spontaneous generalized seizures (SGSs) and interictal activity in CCL17^DTR^ mice. (C) Frequency of SGSs recorded in 16 DT‐treated CCL17^DTR^ mice over a period of 28–30 days. The thick black line shows the average across animals, the gray area the SEM. Seizure activity showed an initial peak 7–9 days after DT, followed by a period of lower activity, which then gradually increased until the end of the recording. This increase was statistically significant (*p* = .00054). (D) Representative maximum intensity projections of combined NeuN (magenta) and glial fibrillary acidic protein (GFAP; green) staining in the CA1 area of coronal hippocampal slices from C57BL/6J (WT) and CCL17^DTR^ mice 30 days after DT injection. Scale bar = 200 μm. (E) The number of CA1 pyramidal neurons and (F) the width of the CA1 stratum radiatum (str. rad.) were significantly reduced in CCL17^DTR^ mice. (G) Granule cell dispersion was not detected in CCL17^DTR^ mice at this time point. (H) The volume occupied by GFAP immunoreactivity, which reflects the extent of reactive astrogliosis, was significantly increased in CCL17^DTR^ mice. *N* = 6 WT and *N* = 5 CCL17^DTR^ mice; *n* = 3 sections/mouse. (I) Representative maximum intensity projections of combined NeuN (magenta) and glial fibrillary acidic protein (GFAP; green) staining in the CA2/3 area of coronal hippocampal slices from C57BL/6J (WT) and CCL17^DTR^ mice 30 days after DT injection. Scale bar = 200 μm. (J, K) Quantification of neuronal density and GFAP immunoreactivity in the hippocampal CA2/3 area (*N* = 3 mice/genotype; *n* = 3 sections/mouse). Boxplots represent median and quartiles. Data were analyzed using Student *t*‐test. ***p < .001. GCL, granule cell layer; str. pyr., stratum pyramidale.

In addition to the significant loss of hippocampal pyramidal neurons, DT treatment increased locomotor activity in CCL17^DTR^ mice compared to both vehicle‐treated CCL17^DTR^ and DT‐treated WT mice (Figure S2D). These findings prompted us to examine the effects of ablating CCL17‐positive neurons on brain activity in vivo. For this, DT‐ and vehicle‐treated CCL17^DTR^ mice were surgically implanted with telemetric EEG transmitters (Figure 2A) 5 days after the first DT injection. EEG analysis revealed that CCL17^DTR^ mice developed SRS in response to DT treatment (Figure 2B), which on average started to emerge on day 9.4 ± 2.7 (range = d6–d15) after the first DT treatment and persisted until the end of the observation period (d30; Figure 2C, *N* = 16 mice). All EEG seizures documented by video monitoring were associated with generalized tonic–clonic convulsions (stage IV–V seizures according to Racine's classification^18^). Nonconvulsive seizures could not be detected with the cortical electrodes. In most mice, seizure frequency showed an initial peak of up to 26 seizures per day at onset, followed by a period of reduced activity, before gradually increasing over the 1‐month observation period (number of seizures on d10–d18: 1.8 ± .29 vs. 3.1 ± .35 on d22–d30, *p* = .0005; Figure 2C). The average number of seizures per day was 2.5 ± 1.1, and the average seizure duration 52.4 ± 10 s. Between seizures, the recorded EEGs revealed interictal spike activity (Figure 2B, upper trace) with a mean frequency of 4 ± 3.2 spikes per minute. Long‐term EEG recordings (until d99 after the first DT injection) in a subset of mice revealed that seizures were persistent, occurring at high frequency up to at least 3 months after the initiation of epilepsy (Figure S3A).

TLE is often associated with the development of HS, a structural pathology characterized by marked neurodegeneration, gliosis, and granule cell dispersion (GCD).^19^ Experimental models of TLE, including the intracortical kainic acid (KA) model, reliably reproduce key features of HS.^20^ Hence, in a next step, we quantified the extent of HS in the dorsal hippocampus of CCL17^DTR^ mice at d30. Immunohistochemistry for the neuronal marker NeuN and the astrocytic marker glial fibrillary acidic protein (GFAP) revealed significant neuronal loss in the CA1 region (Figure 2D,E), shrinkage of the stratum radiatum (Figure 2D,F), and an increase in the area occupied by GFAP (reflecting astrogliosis; Figure 2D,G) in CCL17^DTR^ mice compared to controls. The reduction in the number of pyramidal neurons in the CA2/CA3 region of CCL17^DTR^ mice was close to reaching statistical significance (*p* = .052), and a significant increase in GFAP immunoreactivity was found in that region (Figure 2I–K). GCD could not be detected in CCL17^DTR^ mice at this time point (Figure 2D,H). To determine whether this hippocampal pathology does occur at later stages, we performed morphological analyses in a subset of CCL17^DTR^ mice (*N* = 3) at 2 and 3 months after DT injection. Extensive GCD was detectable in one CCL17^DTR^ mouse at 3 months after DT injection, indicating that HS develops progressively and to a variable degree in CCL17^DTR^ mice (Figure S3B).

In sum, DT‐mediated ablation of hippocampal CCL17‐positive pyramidal neurons induces a robust epileptic phenotype with electrographic and histopathologic alterations resembling human TLE.

### 3.3 Seizure onset in CCL17^DTR^ mice is accompanied by astro‐ and microgliosis

**FIGURE 3:**
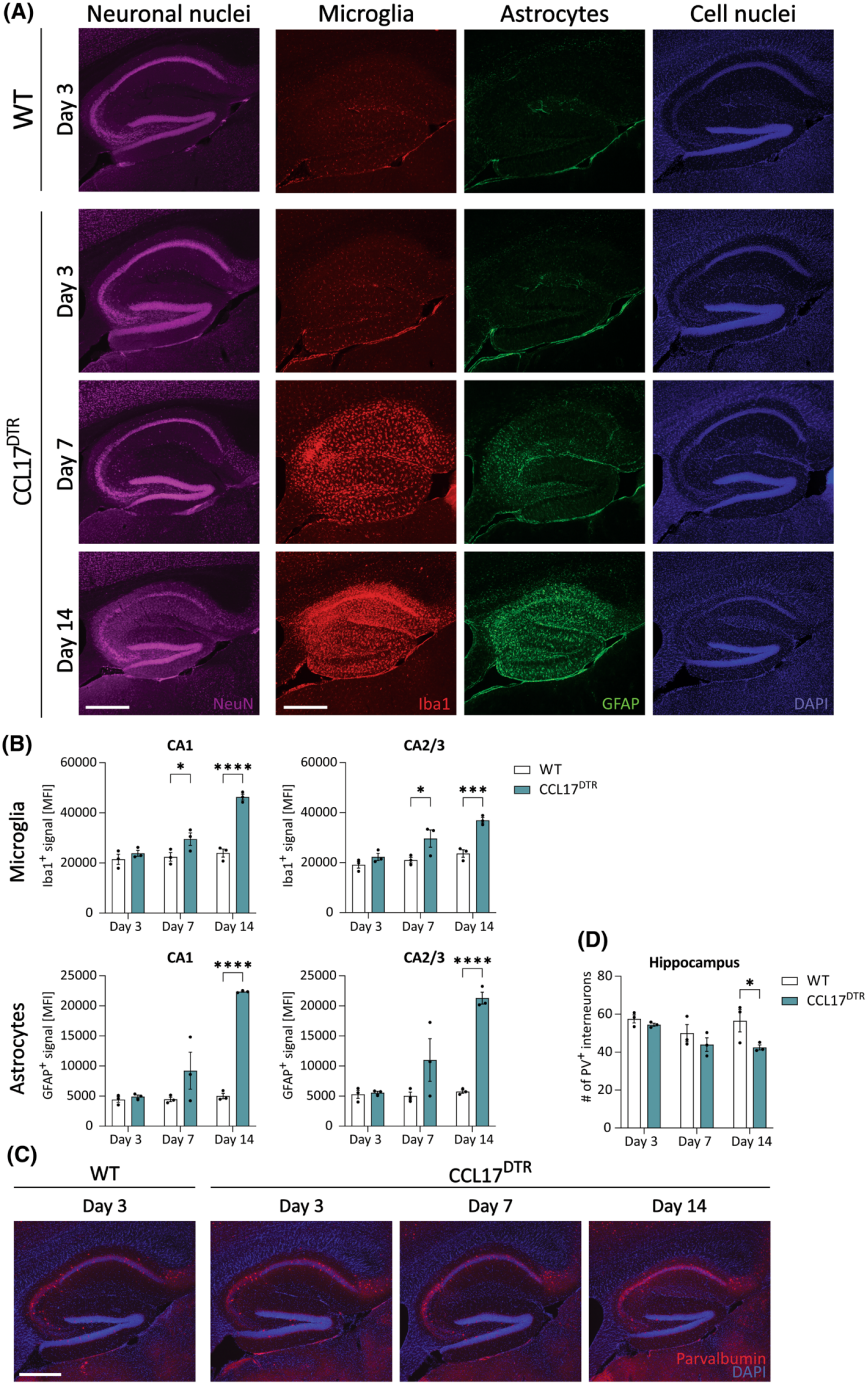
Epileptic seizures are accompanied by extensive gliosis. CCL17^DTR^ and wild‐type (WT) mice received 150 μL phosphate‐buffered saline at day (d) −1 and .4 μg ip DT at d0, d1, and d2. Mice were perfused and brains were isolated at d3, d7, and d14 post‐DT. Images were prepared using epifluorescence microscopy. (A) Forty‐micrometer brain sections were prepared and stained for neuronal nuclei marker NeuN (magenta, first column). In a separate experiment, microglia were stained with Iba1 (red, second column), astrocytes with glial fibrillary acidic protein (GFAP; green, third column) followed by counterstaining for cell nuclei with 4,6‐diamidino‐2‐phenylindole (DAPI; blue, last column). Scale bars (500 μm) apply to all referred panels. Representative images of both experiments are shown. (B) Quantification of Iba1 and GFAP immunoreactivity. Regions of interest were set on CA1 or CA2/CA3 regions. (C) Forty‐micrometer brain sections were stained for the γ‐aminobutyric acidergic interneuron marker parvalbumin (PV) (red) and counterstained with DAPI (blue). Scale bar (500 μm) applies to all panels. (D) Quantification of PV^+^ interneurons in hippocampus. Data were analyzed using one‐way analysis of variance followed by Bonferroni‐adjusted multiple comparisons. *N* = 3 WT and 3 CCL17^DTR^ mice; *n* = 6 sections/mouse for A + B and *n* = 4 sections/mouse for C + D. *p < .05, ***p < .001, ****p < .0001. MFI, mean fluorescence intensity.

Microglia, the principal immune cells of the brain, are known to rapidly react to pathological changes in brain homeostasis, including neuronal stress and degeneration.^21^ To study whether DT‐induced neurodegeneration is accompanied by microglial reactivity, we performed immunohistochemistry for the macrophage marker Iba1 (Figures 3A and S1B). Iba1 staining revealed significant microglial reactivity in the whole hippocampus, including CA1 and CA2/CA3 subfields, at d7 and d14 after DT injection, thus mirroring the pattern of neurodegeneration described in the previous section (Figures 1B,C and 3A,B). Assessment of GFAP staining further indicated a similar progression of astrogliosis (Figures 3A,B and S1B). A more detailed analysis of microglial morphology was performed using MotiQ, an open‐source tool to quantify cell morphology and motility (Figure S4A).^22^ This analysis revealed that microglia in hippocampal CA1 and CA2/CA3 regions retract their processes already at d7 after the start of DT treatment (Figure S4B–D). Moreover, the spanned volume occupied by individual microglial cells was similarly reduced at d7 and d14 following DT treatment in the CA1 region (Figure S4E,F). Interestingly, the microglial surface area was increased at d3 post‐DT in the cortex but normalized at later time points (Figure S4G). In contrast, the microglial surface area was reduced 14 days after DT injection in the hippocampal CA1 region (Figure S4G). Assessment of Iba1 staining in CCL17^DTR^ mice at d5 further suggests that microglia become rapidly reactive in the CA2/CA3 and, partially, in the CA1 region (Figure S5A). Moreover, microglia reactivity appeared to precede astrogliosis (Figures 3B and S5A) and neurodegeneration in CCL17^DTR^ mice (Figure S5B).

Loss of inhibitory interneurons is considered a hallmark of epilepsy and HS.^23^ Targeted ablation of interneurons has been shown to induce SRSs,^24^ and degeneration of PV‐positive interneurons has been described in experimental epilepsy.^25^, ^26^, ^27^ We therefore investigated whether epilepsy induction leads to loss of PV‐positive interneurons in CCL17^DTR^ mice (Figures 3C and S1C). The number of PV‐positive interneurons remained unchanged at d3 and d7 but decreased significantly 14 days after the start of DT treatment (Figure 3D).

Overall, DT‐induced neurodegeneration in CCL17^DTR^ mice is accompanied by astro‐ and microgliosis in the hippocampus. In addition, loss of PV‐positive interneurons was detectable 2 weeks after DT injection.

### 3.4 Neuroinflammatory TNF contributes to epileptogenesis in CCL17^DTR^ mice

**FIGURE 4:**
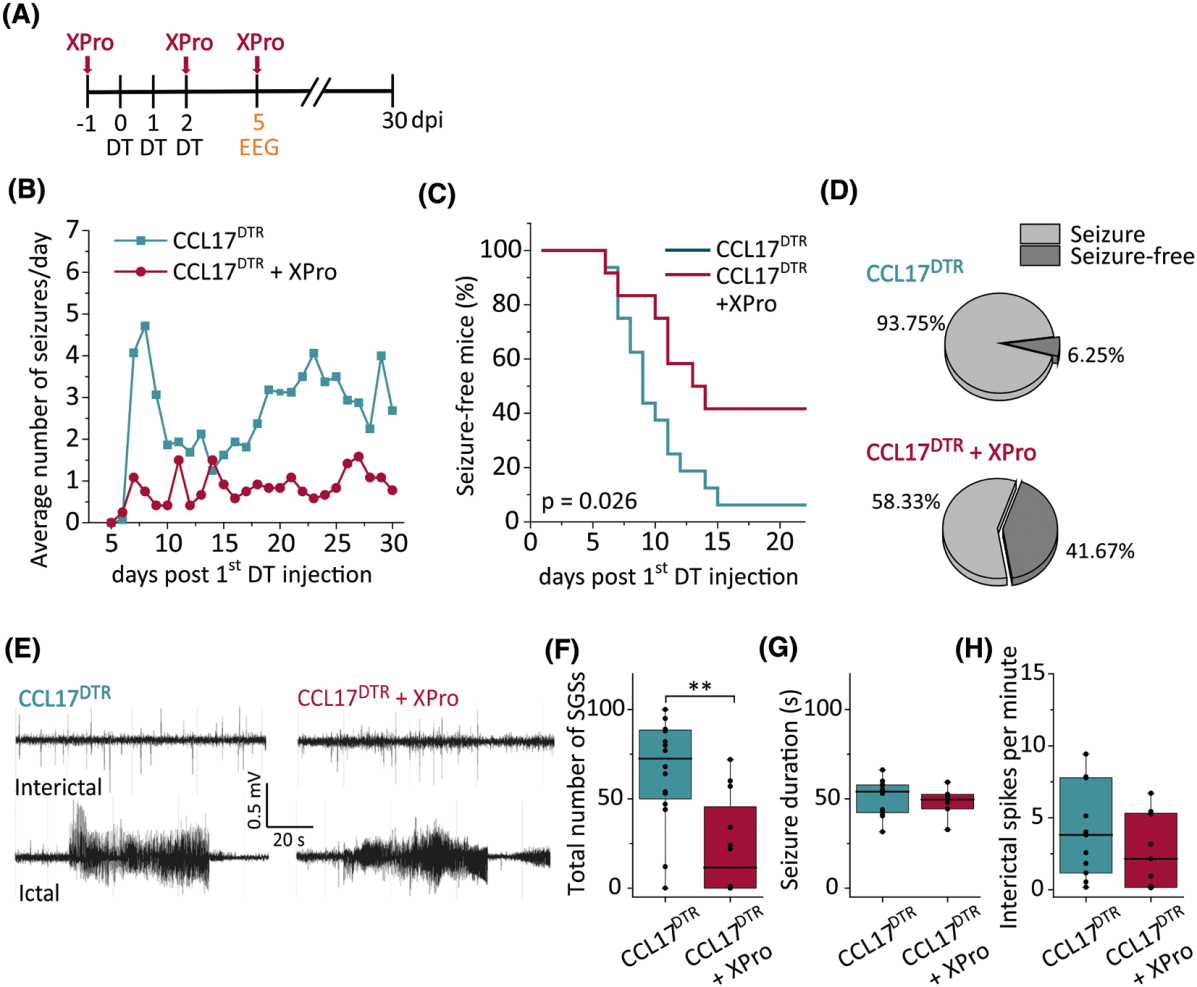
Epileptogenesis in CCL17^DTR^ mice partially depends on proinflammatory soluble tumor necrosis factor signaling. (A) Timeline of the experimental design. Diphtheria toxin (DT)‐treated CCL17^DTR^ mice received either three XPro1595 (XPro; 10 mg/kg ip) or vehicle injections every 72 h starting 1 day before the first DT administration. Animals underwent electroencephalographic (EEG) transmitter implantation on day 5 after the first DT injection to continuously monitor brain activity for 25 days following surgery. (B) Line graph depicting average progression of spontaneous generalized seizure (SGS) activity in CCL17^DTR^ (green) and CCL17^DTR^ + XPro mice (purple) during EEG recording. (C, D) Progression of seizure development and overall percentage of CCL17^DTR^ and CCL17^DTR^ + XPro mice that developed chronic SGSs. (E) Example EEG traces of ictal and interictal activity in vehicle‐ and XPro‐treated CCL17^DTR^ mice. (F, G) XPro treatment significantly reduced the number of SGSs but not their duration. (H) The frequency of interictal spiking during the chronic phase of epilepsy (4th week of recording) was not affected by XPro treatment. *N* = 16 CCL17^DTR^ and *N* = 12 CCL17^DTR^ + XPro mice. Data were analyzed using an independent samples *t*‐test or a log‐rank test. Boxplots represent median and quartiles, with whiskers extending to the highest and lowest values within 1.5 × interquartile range. **p < .01. dpi, days postinjection.

Neuroinflammation is considered a hallmark of epilepsy pathology.^28^ In line with this, the tissue concentration of TNF as determined by enzyme‐linked immunosorbent assay (ELISA) was significantly increased at d5 after the start of DT treatment in the dorsal hippocampus (Figure S5C), thus preceding the emergence of the first SGS and coinciding with the onset of microgliosis in CCL17^DTR^ mice. The proinflammatory cytokine TNF is rapidly released by reactive microglia and exerts proepileptic effects in experimental epilepsy via TNF receptor 1 (TNFR1) signaling.^29^, ^30^ A dominant negative inhibitor of sTNF, XPro1595,^31^ has previously been shown to penetrate the BBB following peripheral administration.^32^ We chose to selectively inhibit sTNF, because it preferentially induces TNFR1 signaling.^33^ XPro1595 or vehicle injections were given three times every third day, starting at d1 prior to the first DT administration (Figure 4A). Interestingly, whereas vehicle‐treated CCL17^DTR^ mice often showed a peak in SGS frequency approximately 7–9 days after the first DT injection, such a peak was not discernible in XPro1595‐treated CCL17^DTR^ mice (Figure 4B). Overall, approximately 58% of XPro1595‐ compared to 94% of vehicle‐treated CCL17^DTR^ mice developed chronic SGSs (Fisher's exact test, *p* = .057; Figure 4C,D). The progression of SGS development was significantly altered by XPro1595 treatment (log‐rank test, *χ* ^2^ = 5, *p* = .026; Figure 4C). Importantly, XPro1595 treatment significantly reduced the total number of SGSs but did not affect SGS duration or the number of interictal spikes during the fourth week of recording in CCL17^DTR^ mice (Figure 4E–H). Of note, in the subset of XPro1595‐treated CCL17^DTR^ mice that did develop SGSs, the seizure burden was nevertheless significantly reduced compared to vehicle‐treated CCL17^DTR^ mice (CCL17^DTR^: 65.5 ± 29.28 [mean ± SD] vs. CCL17^DTR^ + XPro: 38.57 ± 25.28 total seizures, *p* = .047, *N* = 16 CCL17^DTR^ and *N* = 7 CCL17^DTR^ + XPro mice).

To summarize, inhibition of sTNF/TNFR1 signaling attenuates chronic seizure burden in CCL17^DTR^ mice, demonstrating that epileptogenesis following DT‐induced hippocampal neurodegeneration at least partially depends on an inflammatory cascade involving the sTNF/TNFR1 pathway.

### 3.5 Inhibition of sTNF/TNFR1 signaling does not prevent development of HS in epileptic CCL17^DTR^ mice

**FIGURE 5:**
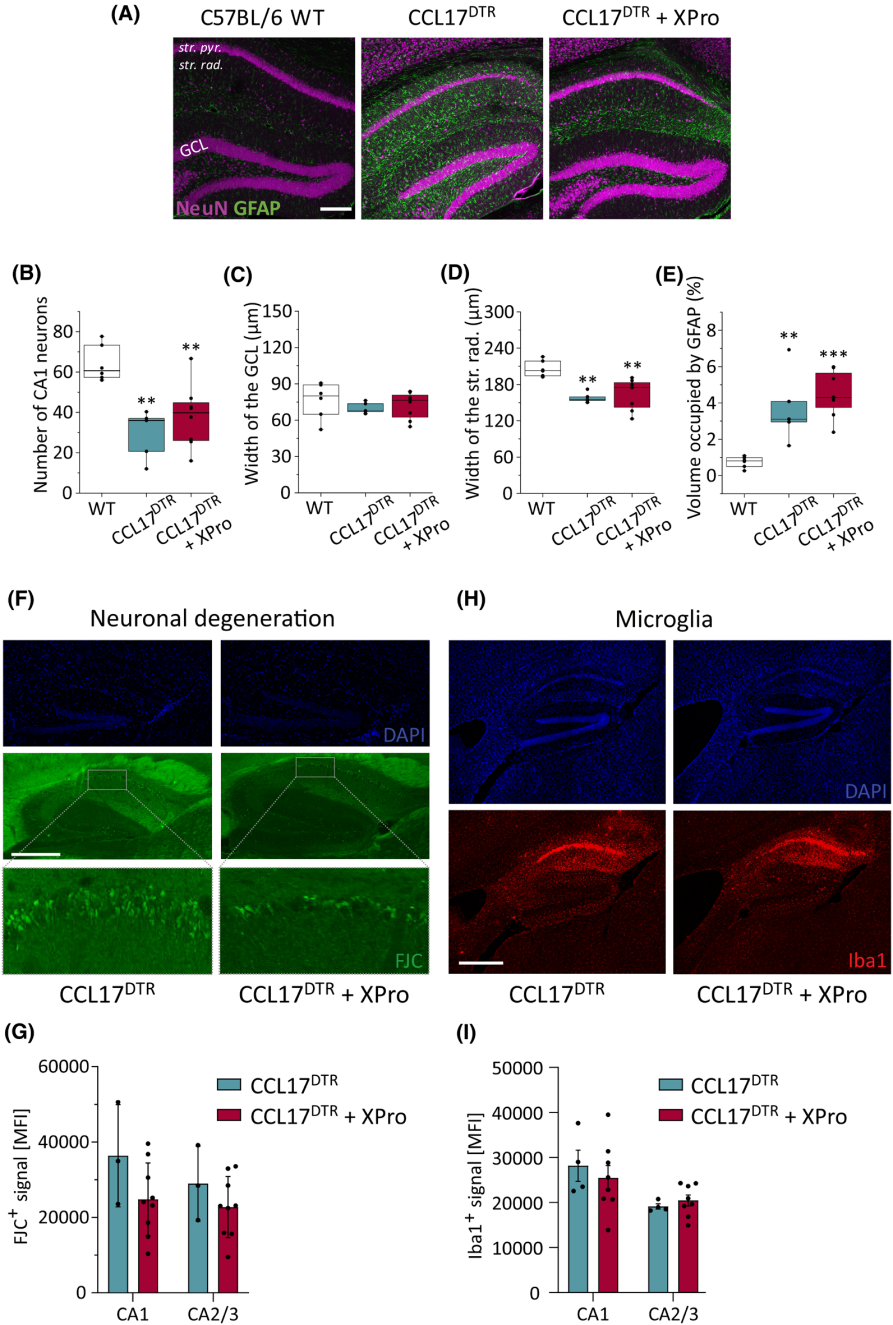
Soluble tumor necrosis factor inhibition does not affect histopathological changes or neuroinflammation in CCL17^DTR^ mice. (A) Representative maximum intensity projections of combined NeuN (magenta) and glial fibrillary acidic protein (GFAP; green) staining in coronal hippocampal slices 1 month after epilepsy induction from diphtheria toxin (DT)‐injected wild‐type C57BL/6J (WT) and CCL17^DTR^ mice pretreated with either vehicle or XPro1595 (XPro). Scale bar = 200 μm. (B–E) XPro treatment did not affect the development of hippocampal sclerosis in CCL17^DTR^ mice. Boxplots represent median and quartiles. *n* = 2–3 sections/mouse from *N* = 6 WT, *N* = 5 CCL17^DTR^, and *N* = 8 CCL17^DTR^ + XPro mice. Data were analyzed using one‐way analysis of variance followed by Tukey's multiple comparisons. (F) Immunofluorescence staining of brain sections at day 30 post‐DT. Sections were analyzed for neuronal degeneration using Fluoro‐Jade C (FJC; green) staining and counterstained for cell nuclei with 4,6‐diamidino‐2‐phenylindole (DAPI; blue). (G) Intensity of FJC^+^ signal was measured in CA1 and CA2/CA3 areas. *N* = 3 CCL17^DTR^ and *N* = 9 CCL17^DTR^ + XPro mice. (H) Sections were stained for microglia (Iba1, red) and counterstained for cell nuclei with DAPI (blue). (I) Intensity of Iba1^+^ signal was measured in CA1 and CA2/CA3 areas. *N* = 3 CCL17^DTR^ and *N* = 7 CCL17^DTR^ + XPro mice. Representative images are shown. Scale bars (500 μm) apply to all panels in their respective subfigure. GCL, granule cell layer; str. pyr., stratum pyramidale; str. rad., stratum radiatum. **p < .01., ***p < .001. MFI, median fluorescence intensity.

As shown in Figure 2, DT treatment induced histopathological changes in the hippocampus of CCL17^DTR^ mice resembling human HS. Interfering with TNFR1 signaling has been shown to be neuroprotective in different models of central nervous system pathology, including experimental epilepsy^30^ and cuprizone‐induced demyelination.^34^ Interestingly, regardless of a reduced seizure burden, XPro1595‐treated CCL17^DTR^ mice displayed a similar extent of CA1 neuron loss (Figure 5A,B), CA1 stratum radiatum shrinkage (Figure 5A,D), and astrocyte reactivity in the dorsal hippocampus 4 weeks after epilepsy induction (Figure 5A,E) compared to vehicle‐treated CCL17^DTR^ mice. GCD was also not observed in XPro1595‐treated mice (Figure 5A,C). Neuronal degeneration was still detectable at d30 in XPro1595‐treated CCL17^DTR^ mice in both the CA1 and the CA2/CA3 region (Figure 5F,G). Moreover, the extent of microglia reactivity in XPro1595‐treated CCL17^DTR^ mice at d30 after DT administration resembled that of vehicle‐treated CCL17^DTR^ mice, indicating seizure‐independent progression of neuroinflammation (Figure 5H,I). Proinflammatory TNF released from reactive microglia impairs interastrocytic GJ coupling, a major astrocyte dysfunction, which is causally involved in the development of mesial TLE with HS (TLE‐HS).^20^, ^29^ Previously, we could show that astrocyte GJ coupling is rapidly and persistently impaired following intracortical KA injection in mice, thereby preceding the onset of the first SGS in this model.^20^ To examine whether functional impairments in astrocytic GJ coupling similarly precede SGS development in CCL17^DTR^ mice, we performed an in situ tracer diffusion assay in hippocampal astrocytes (Figure S5D). However, despite elevated hippocampal TNF concentrations, astrocytic GJ coupling was still intact at d5 after the start of DT administration in CCL17^DTR^ mice (Figure S5D).

Collectively, aberrant sTNF signaling does not appear to be significantly involved in the development of HS in CCL17^DTR^ mice during the first 4 weeks of epileptogenesis. Moreover, astroglial coupling was preserved in DT‐treated CCL17^DTR^ mice at d5 post‐DT, indicating that this is not a major contributing factor to epileptogenesis in CCL17^DTR^ mice.

## 4 DISCUSSION

Neuronal cell loss is a pathological hallmark of both human and experimental TLE,^35^, ^36^ but it remains unclear how it contributes to epileptogenesis and whether it may even be the primary trigger of this process.^4^ There is evidence that neuronal cell death can be both a cause and a consequence of epileptic seizures.^4^, ^37^, ^38^ In electrically or chemically induced mouse models, neuronal cell death occurs often within a few hours, suggesting that the initial stimulation may trigger neurodegeneration, which induces seizures that in turn trigger additional neuronal death.^7^, ^20^ However, astrocytic and microglial changes are often observed prior to neuronal death,^20^, ^29^ calling into question the causal effect of neurodegeneration in epileptogenesis. Many epilepsy cases are thought to be acquired through acute brain injuries, such as trauma, stroke, hypoxia/ischemia, infections, or tumors, which are all associated with neurodegeneration.^39^ Moreover, neurodegenerative diseases such as Alzheimer or Parkinson disease are associated with an increased incidence of epilepsy.^40^ In all these pathologies, however, neurodegeneration is preceded or accompanied by additional molecular and cellular changes, making conclusions about whether neuronal loss represents a contributing factor or a result of epilepsy impossible. Using CCL17^DTR^ mice, which allow sterile and selective ablation of a subpopulation of hippocampal pyramidal neurons upon DT injection, we found that neuronal cell death is sufficient to generate an epileptic phenotype resembling human TLE, including recurrent SGSs and persistent histopathological changes (up to 3 months). Our findings provide the first clear evidence that loss of pyramidal neurons can initiate epilepsy in the absence of other pathological processes.

DT‐mediated ablation of hippocampal pyramidal neurons has been reported previously,^8^, ^10^, ^11^ but no epileptic activity was mentioned in these studies. However, as epilepsy was not the focus of those studies, seizure activity possibly remained unnoticed. On the other hand, our study and the previous ones used different promoters to drive DTR expression, and it may be that only the selective death of CCL17‐positive neurons results in epilepsy. Additionally, reduced CCL17 expression itself may also play a role, as this chemokine modulates synaptic transmission in the hippocampus and exerts neuroprotective effects in experimental brain hemorrhage.^14^, ^41^ This assumption is also supported by the observation that homozygous CCL17^DTR/DTR^ mice had a much higher seizure burden than heterozygous mice, even though the extent of neurodegeneration in response to DT treatment was not different between genotypes (Figure S2). Further work is needed to clarify this point.

DT injection into CCL17^DTR^ mice reliably induces seizures and histopathological changes in the hippocampus, rendering this mouse line a valuable tool for studying molecular mechanisms and treatment options of acquired human TLE caused by neuronal death occurring, for example, in neurodegenerative diseases, stroke, brain trauma, or viral encephalitis. The latter increases the risk of developing human epilepsy by a factor of 16 and often causes medically intractable epilepsy.^42^ Accordingly, viruses such as Theiler murine encephalomyelitis (TMEV) cause limbic seizures and hippocampal damage in animal models that resemble human TLE‐HS,^43^ and it is plausible to assume that virus‐induced death of hippocampal pyramidal neurons is the actual trigger of the disease. Our mouse line might also help to understand the etiology of limbic encephalitis (an autoimmune disorder believed to be the main cause of adult onset TLE‐HS), as hippocampal neuronal loss occurs shortly after its onset.^44^

CCL17^DTR^ mice may reproduce a mechanism of epileptogenesis that occurs in some human epilepsies but is not replicated by commonly used chemical or electrical animal models. The pathogenesis of epilepsy induced in CCL17^DTR^ mice differs significantly from that induced, for instance, by intracortical KA injections.^20^ In the latter model, loss of interastrocytic GJ communication temporally preceded neuronal cell death and spontaneous seizure development, suggesting a causal involvement in epileptogenesis.^20^, ^45^ This pathological astrocytic change is not seen in CCL17^DTR^ mice. Similarly, the loss of PV‐positive interneurons occurs completely and before seizure onset in the KA model,^25^ but only to a small extent and after seizure onset in CCL17^DTR^ mice.

Mechanistically, microglial reactivity and increased TNF levels preceded DT‐triggered neurodegeneration as well as seizure onset (Figures S5 and 2C). Prior to apoptosis, DTR‐expressing neurons may release damage‐associated molecules, such as high‐mobility group box 1 (HMGB1) or adenosine triphosphate, triggering inflammatory responses.^46^ This response could be crucial for seizure development, because depleting sTNF significantly reduced seizure activity. Surprisingly, sTNF inhibition did not affect the extent of neurodegeneration during chronic epilepsy, suggesting that its antiepileptogenic effect is not related to neuronal loss, but instead is attributable to interference with other, cell death‐independent effects of sTNF signaling. Recently, we demonstrated that microglia‐derived TNF rapidly disrupts interastrocytic GJ coupling during KA‐induced status epilepticus.^29^ As mentioned previously, despite elevated hippocampal TNF concentrations, we found no impairment of astroglial coupling before SGS onset in CCL17^DTR^ mice. Notably, the hippocampal TNF concentration in DT‐injected CCL17^DTR^ mice was less than half as high as during KA‐induced acute seizures,^29^ possibly explaining the difference in astroglial coupling efficiency. TNF concentrations might rise further during the progression of epilepsy in DT‐treated CCL17^DTR^ mice, for example, during the peak of SGS activity, and may impair GJ coupling and K^+^ buffering at later time points. Alternatively, increased TNF concentrations in CCL17^DTR^ mice may promote neuronal hyperactivity via other mechanisms, including aberrant glutamate release from astrocytes,^47^ or disturbed astrocytic glutamate uptake.^48^ Moreover, TNF directly affects neurons by increasing α‐amino‐3‐hydroxy‐5‐methyl‐4‐isoxazolepropionic acid receptor and reducing γ‐aminobutyric acid type A receptor trafficking, by enhancing the ratio of excitatory to inhibitory transmission, and modulating synaptic scaling.^49^, ^50^, ^51^ Another notable observation is that seizure activity does not increase over time in XPro1595‐treated mice. In the absence of XPro1595, CCL17^DTR^ mice show a progressive increase in seizure frequency consistent with most forms of human and experimental epilepsy, primarily associated with increasing levels of neuronal damage and scarring, as well as glial and synaptic changes that lower seizure threshold.^52^ This phenomenon is absent in XPro1595‐treated mice, suggesting that high sTNF concentrations at the onset of seizures induce fundamental neuronal and/or astrocytic changes that influence the progression of epilepsy. Interestingly, although XPro1595 exerted strong antiepileptic effects, it did not prevent the development of chronic SGSs in all CCL17^DTR^ mice, indicating that other factors contribute to epileptogenesis in CCL17^DTR^ mice. Several inflammatory mediators exert proepileptic effects, including IL‐1ß,^53^ HMGB1,^54^ and C1q.^55^ Thus, we cannot exclude the possibility that other inflammatory factors are released and contribute to epileptogenesis in CCL17^DTR^ mice. Moreover, the hippocampus is a fine‐tuned cellular network, and ablating a subpopulation of pyramidal neurons is likely to induce axonal sprouting and neural circuit rewiring, forming recurrent excitatory connections that promote seizures, as seen in other models and in human TLE.^56^, ^57^

Finally, ablation of CCL17^+^ neurons induced neuroinflammation in both the CA1 and the CA2/CA3 regions of the hippocampus (Figures 3A,B and S5A), although expression of CCL17 was not observed in the CA2/CA3 area (Figure S6A) and only the CA1 region displayed significant neuronal cell death (Figure 1C). Nevertheless, some FJC‐positive degenerating neurons were also detected in the CA2/CA3 region, such that we cannot exclude that neuronal loss in this region may also contribute to epileptogenesis. Costaining of the CA2 neuron marker PCP4 and the astrocyte marker GFAP confirmed astroglial reactivity in the CA2 region at d7 after DT injection (Figure S6B). Furthermore, microglia reactivity in the CA2/CA3 region was already visible at d5, before any detectable neuronal death (Figure S5A,B). The CA2 area plays a major role in seizure modulation,^58^ but to what degree and how it contributes to epileptogenesis in CCL17^DTR^ mice requires further investigation.

## 5 CONCLUSIONS

In conclusion, we have shown that DTR expression driven by the *Ccl17* promoter is a reliable and efficient approach to induce DT‐mediated pyramidal neurodegeneration in the hippocampus. Importantly, sterile and selective ablation of CCL17‐positive neurons resulted in a robust epileptic phenotype reproducing features of acquired human TLE associated with early neuronal loss. Our results address the long‐standing question of whether neuronal cell death is causative in the development of epilepsy development. We gained important insights into the molecular mechanisms underlying neurodegeneration‐induced epileptogenesis by showing that it partially depends on an inflammatory cascade involving the sTNF/TNFR1 pathway. Hence, CCL17^DTR^ mice provide a powerful tool for mechanistic and translational studies aimed at improving the efficacy and tolerability of antiseizure medication.

## AUTHOR CONTRIBUTIONS

*Conceptualization:* Judith Eberhard, Lukas Henning, Lorenz Fülle, Julia Kenzler, Heike Weighardt, Peter Bedner, Christian Steinhäuser, and Irmgard Förster. *Methodology:* Judith Eberhard, Lukas Henning, Lorenz Fülle, Julia Kenzler, Frederic Brosseron, Matthias Lochner, and Peter Bedner. *Software:* Lorenz Fülle. *Validation:* Judith Eberhard, Lukas Henning, Lorenz Fülle, Matthias Lochner, and Peter Bedner. *Formal analysis:* Judith Eberhard, Lukas Henning, Lorenz Fülle, Konrad Knöpper, and Peter Bedner. *Investigation:* Judith Eberhard, Lukas Henning, Lorenz Fülle, Konrad Knöpper, Jana Böhringer, Lea Hänschke, Frederike J. Graelmann, Julia Kenzler, Frederic Brosseron, Stefanie Eyerich, Matthias Lochner, and Peter Bedner. *Resources:* Ana I. Domingos, Christian Steinhäuser, and Irmgard Förster. *Data curation:* Judith Eberhard, Lukas Henning, Lorenz Fülle, Konrad Knöpper, and Peter Bedner. *Writing—original draft preparation:* Judith Eberhard, Lukas Henning, Peter Bedner, Christian Steinhäuser, and Irmgard Förster. *Writing—review and editing:* Judith Eberhard, Lukas Henning, Peter Bedner, Christian Steinhäuser, and Irmgard Förster. *Visualization:* Judith Eberhard, Lukas Henning, Lorenz Fülle, Konrad Knöpper, and Peter Bedner. *Supervision:* Michael T. Heneka, Heike Weighardt, Peter Bedner, Christian Steinhäuser, and Irmgard Förster. *Funding acquisition:* Christian Steinhäuser and Irmgard Förster.

## CONFLICT OF INTEREST STATEMENT

None of the authors has any conflict of interest to disclose. We confirm that we have read the Journal's position on issues involved in ethical publication and affirm that this report is consistent with those guidelines.

## Supporting information


Data S1.

Figure S1.

Figure S2.

Figure S3.

Figure S4.

Figure S5.

Figure S6.

## Data Availability

The data that support the findings of this study are available from the corresponding author upon reasonable request.
