# Supplementary material for: Ablation of CCL17‐positive hippocampal neurons induces inflammation‐dependent epilepsy
Source: Epilepsia. 2024 Nov 28;66(2):554–68. doi: 10.1111/epi.18200 (PMC11827734; doi:10.1111/epi.18200)
Supplement: Supplementary file 1 — Data S1. [file EPI-66-554-s001.pdf]

Supporting Information Materials & Methods

for the manuscript

Ablation of CCL17-positive hippocampal neurons induces inflammation-dependent epilepsy

1. Animals

1.1 Animal husbandry

All mice were bred under specific pathogen free conditions in the Genetic Resources Center (GRC) of the Life & Medical Sciences (LIMES) Institute in Bonn, Germany. Mice were kept in individually ventilated animal cages and maintained under specific pathogen-free conditions (12 h/12 h light-dark cycle, 22°C), with *ad libitum* access to food and water. All experiments were performed using male or female 8–12 weeks old C57BL/6JRCC WT or transgenic mice. All experiments were performed in accordance with EU and local governmental regulations. Experiments were approved by the North Rhine–Westphalia State Agency for Nature, Environment and Consumer Protection (84-02.04.2015.A393, 84-02.04.2016.A409, 81-02.04.2020.A420, 81-02.04.2021.A426).

1.2 Number of animals

**Figure**	**Number of animals used (N)**	**Number of sections/cells analyzed per animal (n)**
Figure 1	WT = 4-5, CCL17^{DTR} = 5-6	n = 1 section
Figure 2B+C	CCL17^{DTR} = 16	-
Figure 2D-H	WT = 6, CCL17^{DTR} = 5	n = 3 sections
Figure 2I-K	WT = 3, CCL17^{DTR} = 3	n = 3 sections

Figure 3A-C	WT = 3, $CCL17^{DTR}$ = 3	n = 6 sections
Figure 3D	WT = 3, $CCL17^{DTR}$ = 3	n = 4 sections
Figure 4	$CCL17^{DTR}$ = 16, $CCL17^{DTR}$ + XPro = 12	-
Figure 5B-E	WT = 6, $CCL17^{DTR}$ = 5, $CCL17^{DTR}$ + XPro = 8	n = 2-3 sections
Figure 5F+G	$CCL17^{DTR}$ = 3, $CCL17^{DTR}$ + XPro = 9	n = 1 section
Figure 5H+I	$CCL17^{DTR}$ = 3, $CCL17^{DTR}$ + XPro = 7	n = 1 section
Figure S1A+B	WT = 3	n = 6 sections
Figure S1C	WT = 3	n = 4 sections
Figure S2A+C	WT = 3, $CCL17^{DTR}$ = 3 (PEGyDT)	n = 1 section
Figure S2B+C	$CCL17^{DTR}$ = 3, $CCL17^{DTR/DTR}$ = 3 (DT)	n = 1 section
Figure S2D	WT (DT) = 3, $CCL17^{DTR}$ (PBS) = 3, $CCL17^{DTR}$ (DT) = 3	-
Figure S3	$CCL17^{DTR}$ = 3	n = 1 section
Figure S4	WT = 2, $CCL17^{DTR}$ = 3	n = 4 cells/region
Figure S5A+B	WT = 3, $CCL17^{DTR}$ = 3	n = 1 section
Figure S5C	$CCL17^{DTR}$ = 6 (vehicle), $CCL17^{DTR}$ = 6 (DT)	n = 2 hippocampi per animal
Figure S5D	$CCL17^{DTR}$ = 3 (vehicle), $CCL17^{DTR}$ = 3 (DT)	n = 14-15 cells

Figure S6A	CCL17^{EGFP} = 4	n = 1 section
Figure S6B	WT = 1, CCL17^{DTR} = 2	n = 1 section

1.3. Generation of CCL17-DTR mice

Homozygous CCL17-EGFP mice (CCL17$^{E/E}$) were generated as previously described (1). For generation of CCL17^{DTR} mice expressing the simian DTR (2, 3), the DTR cDNA (3) and a neomycin cassette flanked by flippase recognition target (FRT)-recombination sites were inserted in the second exon of the *Ccl17* gene similarly to the generation of CCL17Cre mice (4). Homologously recombined embryonic stem cell clones (E14.1) were detected by Southern blot and the neomycin-resistance cassette was removed from the targeted *Ccl17* locus by flippase (FLP) recombinase expression. After germline transmission, mice were backcrossed to the C57BL/6JRCC background for more than eight generations. Both homozygous and heterozygous CCL17^{DTR} mice (CCL17$^{DTR/DTR}$ and CCL17^{DTR} mice, respectively) were used as indicated.

1.4 Assessment of locomotor behavior

Mice were positioned in an empty cage and a 5 min video of each mouse was recorded with a custom-build Raspberry Pi Camera System using a software coded by L. Fülle. In each video the first 60 s, when mice accustomed to the new environment, were excluded. Videos were processed in Any Video Converter (Anvsoft Inc.) and analyzed with Fiji.

2. EEG recordings

2.1. Implantation of telemetric EEG transmitters

Two drill holes were made at ~3 mm posterior to bregma and 1.5 mm lateral from midline for the insertion of two monopolar leads ~1 mm deep into the cortex to detect electrographic seizures. Leads were subsequently fixed to the skull using superglue and covered with dental cement (Paladur®, Kulzer GmbH, Hanau, Germany). Telemetric transmitters (TA10EA-F20 or TA11ETA-F10; Data Sciences International (DSI), St. Paul, MN, USA) were implanted subcutaneously into the right abdominal region immediately after lead insertion. Subsequently, the scalp and abdominal incisions were sutured and anesthesia stopped using atipamezol (Antisedan, Orion Pharma, Hamburg, Germany, 300 mg/kg, i.p.). To reduce pain, mice were injected for three consecutive days with carprofen (Rimadyl, Pfizer, Karlsruhe, Germany, 5 mg/kg, i.p.). Additionally, 0.25% Enrofloxacin (Baytril, Bayer, Leverkusen, Germany) was administered *via* the drinking water to minimize the risk of infection. Following surgery, mice were returned to clean cages and placed on individual radio receiving plates (RPC-1; Data Sciences International, New Brighton, MN, USA), which capture data signals from the transmitter and transfer them to a computer running Ponemah software (Version 5.2, Data Sciences International) to convert the digital output of the receiver into a calibrated analog output. For a subset of animals, EEG recordings were continued up until 99 d after the start of DT treatment.

2.2. EEG data analysis

Seizure duration and spike numbers were determined using the spike train analysis tool implemented in NeuroScore™ based on the following criteria: threshold value = 7.5 x SD of the baseline (i.e., activity during artifact- and epileptiform-free epochs) – 1,000 µV, spike duration = 0.1 – 50 ms, spike interval = 0.1 – 2.5 s, minimum train duration = 30 s, train join interval = 1 s, minimum number of spikes = 50. Prior to spike

analysis, recordings were high pass filtered at 1 Hz. The number of artifacts included into spike counts was minimized by repeating the spike train analysis with a threshold ranging from the maximum spike amplitude observed – 1000 µV and subsequently subtracting that value from the value obtained during initial spike quantification (i.e., 7.5 x SD of the baseline – 1,000 µV). The number of spontaneously generalized seizures was determined manually by two experienced experimenters.

3. Whole-cell patch clamp recordings and biocytin-loading of astrocytes

Brains were quickly removed and put into ice cold preparation solution containing (in mM): 87 NaCl, 2.5 KCl, 1.25 NaH_2PO_4, 25 $NaHCO_3$, 7 $MgCl_2$, 0.5 $CaCl_2$, 25 glucose, 75 sucrose, equilibrated with carbogen (5% CO_2/95% O_2, pH 7.4). Brains were cut into 200 µm-thick coronal slices using a vibratome (VT1000S, Leica, Wetzlar, Germany). Slices were subsequently stored in preparation solution for 15 min at 35°C and then transferred to aCSF containing (in mM): 126 NaCl, 3 KCl, 2 $MgSO_4$, 2 $CaCl_2$, 10 glucose, 1.25 NaH_2PO_4, 26 $NaHCO_3$ gassed with carbogen. To facilitate the identification of astrocytes in the tissue, slices were incubated in aCSF containing Sulforhodamine 101 (1 µM, Sigma Aldrich, S7635, Steinheim, Germany; incubation 20 min, 35°C) (6). Next, slices were transferred to aCSF and kept at room temperature (RT) for the duration of the experiment. Prior to starting the recordings, slices were transferred to a recording chamber and constantly perfused with aCSF. Patch pipettes fabricated from borosilicate capillaries with a resistance of 3-5 MΩ were filled with a solution containing (in mM): 130 K-gluconate, 1 $MgCl_2$, 3 Na_2-ATP, 20 HEPES, 10 EGTA and biocytin (0.5%, #B4261, Sigma Aldrich) (pH 7.2, 280-285 mOsm). Astrocytes were identified by SR101 fluorescence, their characteristic morphology including a small soma size, a passive current-voltage relationship and a resting

membrane potential close to the equilibrium potential of K^+ (7). Current signals were amplified (EPC 8, HEKA Electronic, Lambrecht, Germany), filtered at 3 or 10 kHz, and sampled at 10 or 30 kHz (holding potential –80 mV). Online analysis was conducted using TIDA 5.25 acquisition and analysis software for Windows (HEKA) and Igor Pro 6.37 software (WaveMetrics, Lake Oswego, OR, USA). Liquid junction potentials were corrected online. Only recordings matching the following criteria were included into the analysis: i) resting potential negative to -60 mV, ii) membrane resistance ≤ 10 MΩ and iii) series resistance ≤ 20 MΩ. After recording, slices containing a biocytin-filled astrocyte were stored in 4% PFA-containing PBS solution overnight at 4°C and subsequently put into PBS and stored at 4°C until immunohistochemistry.

4. Immunohistochemistry

4.1. Perfusion of mouse brains

Animals that underwent EEG recordings were deeply anesthetized by i.p. injection with 100-120 µl of a solution containing 80 mg/kg ketamine (WDT) and 0.3 mg/kg medetomidine (CP-Pharma). A solution containing 300 mg/kg ketamine (Vetoquinol GmbH, Ismaning, Germany) and 20 mg/kg xylazine hydrochloride (WDT) was administered i.p. to anaesthetize animals that were not subjected to EEG recordings. Hind paw reflexes were checked to ensure deep anesthesia. Next, transcardial perfusion was performed using ice-cold PBS (25-30 ml) followed by 4% ice-cold PFA in PBS (25-30 mL). Brains were removed and stored overnight in 4% PFA-containing solution and were subsequently transferred into PBS at 4°C until slicing. Brain sections were cut into 40 µm coronal or sagittal slices using a Leica VT1200S or VT1000 vibratome (Leica Microsystems). Parasagittal brain sections were prepared by first separating the cerebral hemispheres along the longitudinal plane. Next, sagittal

sections were cut from right brain hemispheres (for Figure 3, also left brain hemispheres) and stored in 2 mL Eppendorf tubes containing PBS with 0.05%-0.09% sodium azide for long-term storage.

4.2. Histological staining

Immunohistochemistry was performed using free-floating slices kept in 24-well plates. Only slices from the dorsal hippocampus were used for staining. For membrane permeabilization and blocking of unspecific epitopes, slices were incubated (2 h, RT) with 0.5% Triton X-100 (or 2% for staining of biocytin-filled astrocytes) and 10% normal goat serum (NGS) in PBS. Slices were subsequently incubated overnight with primary antibody solution containing PBS on a shaker at 4°C. The following primary antibodies were applied: rabbit anti-GFAP (1:500, DAKO, Z0334, Hamburg, Germany or 1:500, Thermo Fisher Scientific, 130300), mouse anti-NeuN (1:300, Merck Millipore, MAB377, Darmstadt, Germany), rabbit anti-IBA1 (1:500, Wako Chemicals, #019-19741, Neuss, Germany), mouse anti-parvalbumin (1:1000, Merck Millipore, MAB1572), rabbit anti-s100β (1:500, Abcam, ab41548, Berlin, Germany), rabbit anti-PCP4 (1:200, Proteintech, 14705-1-AP, Rosemont, USA). On the following day, slices were washed three times with PBS for 10-15 min each, followed by incubation with secondary antibodies conjugated with Alexa Fluor® 488 (1:500, Thermo Fisher Scientific, S32354), Alexa Fluor® 594 (1:500, Life Technologies, A11012) or Alexa Fluor® 647 (1:500, Invitrogen, Karlsruhe, Germany or 1:500, Biolegend, 405416) or streptavidin-conjugated Alexa Fluor® 647 for labeling of biocytin (1:600, Invitrogen, Karlsruhe, Germany) in PBS (2% NGS, 1.5 - 2h, RT). For staining of NeuN, slices were incubated with goat anti-mouse biotin (1:500, Dianova, AB_2338557, Hamburg, Germany; 2h, RT) prior to incubation with streptavidin-conjugated Alexa Fluor® 488

antibody (1:300, Thermo Fisher Scientific, S32354; 1 h, RT). Alternatively, slices were incubated with goat anti-mouse Alexa Fluor™ 647 (1:500, Thermo Fisher Scientific, A21237). After washing slices three times with PBS (10-15 min), nuclear staining with Hoechst (1:200, diluted in ddH_2O, 10 min RT) or DAPI (1:1000 in PBS, 5 min RT) was performed. A final washing step (3x PBS, 5 min each) was performed and slices were mounted with Aquapolymount (Polysciences, Heidelberg, Germany) or 70 µl Mowiol (Roth®, Karlsruhe, Germany) on objective slides and covered with cover slips. Slides were stored at 4°C for at least 24 h before microscopic imaging.

4.3. Fluoro Jade C staining

For visualization of neuronal degeneration, brain sections were stained with Fluoro Jade C (FJC) (AG325, Sigma-Aldrich), according to a modified protocol from (8). All staining steps were performed at RT. Brain sections from the dorsal hippocampus were washed three times with 500 µL ddH_2O for 10 min on an orbital shaker and incubated in potassium permanganate solution (0.06% potassium permanganate in ddH2O) for 10 min. Staining was performed in FJC working solution (0.0001% FJC in ddH_2O) for 10 min and washed three times in ddH2O for 10 min. Subsequently, the sections were air-dried and mounted on coverslips with DPX mounting medium. Mounted sections were air-dried for at least 24h prior to microscopy.

4.4. Imaging

4.4.1 Confocal microscopy

Imaging was performed either using a Zeiss® LSM 780 (Carl Zeiss Microscopy GmbH, Jena, Germany) or a Leica SP8 (Hamburg, Germany) confocal laser scanning microscope at 8 bit using 10x (numerical aperture (NA): 0.4) or 20x (NA: 0.75)

objectives. Image resolution was set at 1,024 x 1,024 pixels recorded at a speed of 400 Hz, with a pinhole size of 1 airy unit (AU) and a digital zoom of 1 (hippocampal sclerosis) or 1.2 (GJ coupling). Standard photomultiplier tubes were used for detection of fluorescent signals and laser and detector settings were applied equally to all images acquired. Z-stacks were recorded at 2 µm intervals.

4.4.2 Epifluorescence microscopy

Epifluorescence imaging was performed using a BZ-9000E Keyence microscope (Keyence, Montabaur, Germany) with 10x (NA: 0.20) or 20x (NA: 0.75) objectives. Image resolution in 8bit monochrome, multidimensional photo mode was set at 1360x1024 pixels. Multidimensional images were generated using the built-in functions from BZ-II Viewer and BZ-II Analyzer Software (Keyence, Montabaur, Germany). Excitation times were applied equally to all images acquired per experiment or analysis. Z-stacks were recorded at 1 µm intervals.

5. Image analysis and quantification

<u>5.1. Hippocampal sclerosis</u>

The extent of HS was determined based on the quantification of three parameters: i) extent of granule cell dispersion (GCD) in the dentate gyrus (DG), ii) shrinkage of the CA1 *stratum radiatum* and iii) the number of pyramidal neurons in CA1 *pyramidal layer*. The parameters were estimated in maximum intensity projections MIPs (1,163 x 1,163 x 40 µm^3). GCD quantification was performed as described previously (Henning et al., 2023). Briefly, the width of the granule cell layer (GCL) was determined at four positions indicated as T1–T4. T1 and T2 were measured along a vertical line connecting the upper and lower cell layers of the DG, T3 and T4 at a distance halfway

between the vertical line and the tip of the hilus. The average of the four values was used as an estimation of GCD. Shrinkage of the *stratum radiatum* was determined by drawing a vertical line connecting the pyramidal and molecular layer, above the peak of the GCL. The length of the vertical line served as an indication of the remaining width of the *stratum radiatum*. The degree of astrogliosis was determined by quantifying the area occupied by the GFAP signal in individual regions of interest (ROIs) within each image. Total volume occupied by the GFAP signal was subsequently derived by summing up the area calculated across all focal planes and was normalized to the volume of the ROI prior to statistical analysis (9). Astrogliosis, GCL,*stratum radiatum* width and the number of pyramidal neurons in the CA2/3 were quantified using Fiji software. Finally, the number of pyramidal neurons in the CA1 region was determined using the automated spot detection algorithm implemented in IMARIS 8.0 within a 360 x 120 x 40 μm^3 ROI placed within the CA1 pyramidal layer just above the peak of the GCL.

5.2. Quantification of microglia morphology

Microglia morphology was analyzed using MotiQ, a plugin for Fiji (10), allowing semi-automated analysis of morphological properties of microglia (11). Analysis was adapted from (12). Briefly, representative single microglia cells per animal were cropped out of Z-stacks and converted to binary images applying the “MinError” threshold implemented in Fiji. Particles smaller than 250 voxels were removed and surface area, cell volume, spanned volume and ramification of the single cell were automatically reconstructed. The ramification index describes the ratio of the surface area of a cell to the surface area of a sphere containing the same volume as the cell (11). A Gauss filter with sigma = 2.0 was applied prior to acquisition of skeletal

parameters (number of branches and tree length). Analysis of generated skeletons was performed using plugins by (13). All parameters examined are described in detail in (11).

5.3. Quantification of Iba1 and GFAP fluorescence intensity

Digital image analysis was performed using built-in functions for ImageJ (Fiji). Mean fluorescence was analyzed in manually selected areas of the hippocampus (CA1 and CA2/CA3 areas). Background noise was subtracted prior to image analysis.

5.4. Quantification of the number of FJC-positive cells

A custom-written analysis script (FIJI) was used to determine the number of FJC-positive cells in DTR mice at different time points after DT injection. Images from DT-treated WT mice were analyzed to serve as a negative control. Individual ROIs of the CA1 and CA2/CA3 regions were defined in epifluorescence images of the whole hippocampus. Images were background subtracted using the rolling ball algorithm implemented in FIJI (radius = 50 pixel), median filtered (radius = 3 pixel) and subsequently binarized using the *Triangle* threshold. FJC cell number was quantified using the *analyze particles* function (particle size = 75 – 5000 pixel, circularity 0.15 – 1) and normalized to the total volume of the respective ROI to account for differences in ROI size. Data is depicted as the number of FJC-positive cells per mm^{-3}. For Supplementary figure 1, number of FJC positive cells was counted manually.

5.5. Coupling efficiency of biocytin-filled astrocytes

Coupling efficiency was determined by manual counting of biocytin$^+$ cells using the cell counter plugin of Fiji. Another observer blinded to the experimental conditions

recounted images of biocytin-filled astrocytes, and cell counts were subsequently averaged across both counts prior to statistical analysis.

5.6. Quantification of parvalbumin (PV)-positive interneurons

PV-positive interneurons localized in the hippocampal area were counted manually by three blinded experimenters from one image per animal.

6. Quantification of hippocampal TNF concentrations

6.1. Tissue preparation and biochemical extraction of hippocampal protein

CCL17^{DTR} mice were transcardially perfused. Dorsal hippocampi were quickly removed, weighed and flash frozen in liquid nitrogen before storage at -80°C until biochemical extraction. Frozen hippocampi were homogenized in homogenization buffer (1x PBS, 5 mM NaF, 20 mM pyrophosphate, 1x protease/phosphatase inhibitor; Cell Signaling, #5872, Frankfurt am Main, Germany) using a Precellys device (Bertin Instruments, Darmstadt, Germany) for 2x 15 s at 5,000 rpm. Next, an equal volume of 2x RIPA buffer (50 mM Tris, 150 mM NaCl, 2% NP-40, 1% NaDOC, 0.2% SDS) was added to the sample, sonicated for 10 s and incubated on ice for 30 min. The homogenate was centrifuged for 30 min at 100,000*g* and the supernatant collected. Total protein concentration in the supernatants was determined using a bicinchoninic acid (BCA) assay (Thermo Fisher Scientific, Bremen, Germany).

6.2. ELISA

For quantification of hippocampal TNF concentrations an electrochemiluminescence ELISA (Meso Scale Discovery) was performed. Each sample was measured in duplicate and the average was normalized to the weight of hippocampal tissue and

expressed as pg/µg tissue. Two vehicle-treated CCL17^{DTR} mice had TNF levels below the detection limit of the ELISA assay and were therefore excluded from statistical analysis.

7. Statistical analysis

Prior to statistical analysis, data were checked for normality by inspection of histograms and Q–Q plots as well as by applying a Shapiro–Wilk test. Levene's test was performed to check for homogeneity of variance between groups. In case of a significant deviation from normality, the non-parametric equivalent statistical test was performed. For comparison of two independent groups, an independent samples t-test or a Wilcoxon-rank sum test was applied. More than two groups were analyzed using one-way ANOVA followed by Bonferroni´s or Tukey's post-hoc test to adjust for multiple comparisons, or a Kruskal-Wallis test was conducted. For multifactorial data, statistical differences were analyzed by Two-way ANOVA. To analyze DT-mediated cell death a linear mixed-model regression of Tukey-transformed data ($\lambda = 0.25$) was performed followed by a contrasts analysis, to specifically compare the extent and progression of the number of FJC-positive cells in CCL17^{DTR} mice in the hippocampal CA1 and CA2/CA3, respectively. Kaplan–Meier estimates were compared using a log-rank test.

Literature Cited

1. Alferink J, Lieberam I, Reindl W, Behrens A, Weiss S, Hüser N et al. Compartmentalized production of CCL17 in vivo: strong inducibility in peripheral dendritic cells contrasts selective absence from the spleen. J Exp Med 2003; 197(5):585–99.

2. Saito M, Iwawaki T, Taya C, Yonekawa H, Noda M, Inui Y et al. Diphtheria toxin receptor-mediated conditional and targeted cell ablation in transgenic mice. Nat Biotechnol 2001; 19(8):746–50.

3. Jung S, Unutmaz D, Wong P, Sano G-I, los Santos K de, Sparwasser T et al. In vivo depletion of CD11c+ dendritic cells abrogates priming of CD8+ T cells by exogenous cell-associated antigens. Immunity 2002; 17(2):211–20.

4. Köhler T, Reizis B, Johnson RS, Weighardt H, Förster I. Influence of hypoxia-inducible factor 1α on dendritic cell differentiation and migration. Eur J Immunol 2012; 42(5):1226–36.

5. Deshpande T, Li T, Henning L, Wu Z, Müller J, Seifert G et al. Constitutive deletion of astrocytic connexins aggravates kainate-induced epilepsy. Glia 2020; 68(10):2136–47.

6. Kafitz KW, Meier SD, Stephan J, Rose CR. Developmental profile and properties of sulforhodamine 101--Labeled glial cells in acute brain slices of rat hippocampus. Journal of Neuroscience Methods 2008; 169(1):84–92.

7. Bedner P, Jabs R, Steinhäuser C. Properties of human astrocytes and NG2 glia. Glia 2020; 68(4):756–67.

8. Schmued LC, Stowers CC, Scallet AC, Xu L. Fluoro-Jade C results in ultra high resolution and contrast labeling of degenerating neurons. Brain Res 2005; 1035(1):24–31.

9. Henning L, Antony H, Breuer A, Müller J, Seifert G, Audinat E et al. Reactive microglia are the major source of tumor necrosis factor alpha and contribute to astrocyte dysfunction and acute seizures in experimental temporal lobe epilepsy. Glia 2023; 71(2):168–86. Available from: URL: https://onlinelibrary.wiley.com/doi/10.1002/glia.24265.

10. Schindelin J, Arganda-Carreras I, Frise E, Kaynig V, Longair M, Pietzsch T et al. Fiji: an open-source platform for biological-image analysis. Nat Methods 2012; 9(7):676–82.

11. Hansen JN, Brückner M, Pietrowski MJ, Jikeli JF, Plescher M, Beckert H et al. MotiQ: an open-source toolbox to quantify the cell motility and morphology of microglia. Mol Biol Cell 2022; 33(11):ar99.

12. Fülle L, Offermann N, Hansen JN, Breithausen B, Erazo AB, Schanz O et al. CCL17 exerts a neuroimmune modulatory function and is expressed in hippocampal neurons. Glia 2018; 66(10):2246–61.

13. Arganda-Carreras I, Fernández-González R, Muñoz-Barrutia A, Ortiz-De-Solorzano C. 3D reconstruction of histological sections: Application to mammary gland tissue. Microsc Res Tech 2010; 73(11):1019–29.

14. R: A Language and Environment for Statistical Computing. Version 4.0.5. Vienna, Austria; 2021. Available from: URL: https://www.R-project.org/.
