# Supplementary material for: Ablation of CCL17‐positive hippocampal neurons induces inflammation‐dependent epilepsy
Source: Epilepsia. 2024 Nov 28;66(2):554–68. doi: 10.1111/epi.18200 (PMC11827734; doi:10.1111/epi.18200)
Supplement: Supplementary file 2 — Figure S1. [file EPI-66-554-s006.pdf]

Figure S1

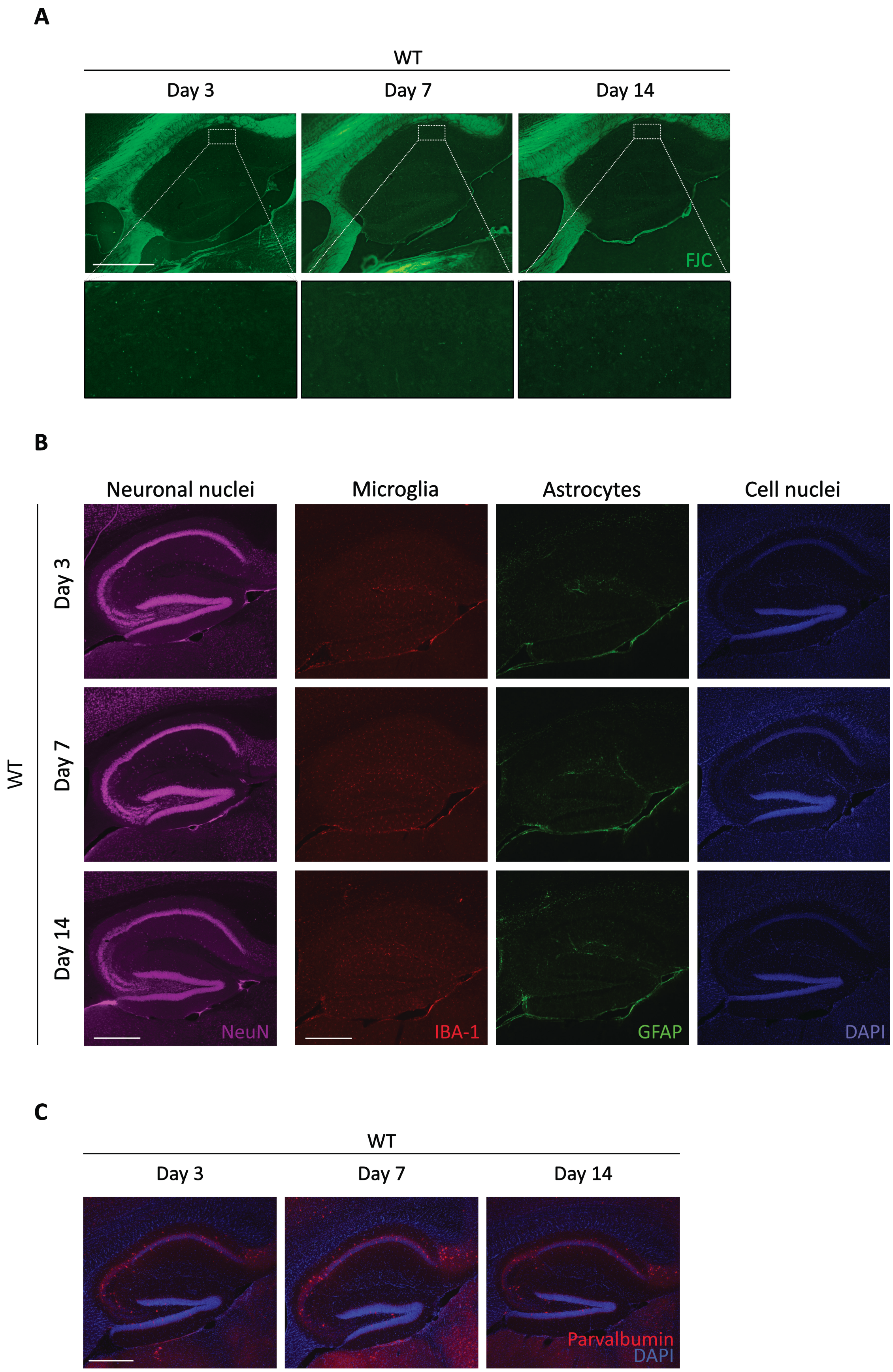
A
WT
Day 3
Day 7
Day 14
FJC
B
Neuronal nuclei
Microglia
Astrocytes
Cell nuclei
WT
Day 3
Day 7
Day 14
NeuN
IBA-1
GFAP
DAPI
C
WT
Day 3
Day 7
Day 14
Parvalbumin
DAPI

Figure S1 | DT-treated WT animals do not develop neuroinflammation or neurodegeneration.

WT mice received 150 µL PBS at day -1 and 0.4 µg DT i.p. at d0, 1, and 2. Mice were perfused in situ and brains were isolated at d3, 7 and 14. (A) Forty µm brain sections were prepared and degenerating neurons detected by Fluoro Jade C labeling (FJC, green). (B) Forty µm brain sections were stained for neuronal nuclei (NeuN, magenta), microglia (Iba1, red), astrocytes (GFAP, green) and counterstained for cell nuclei (DAPI, blue). (C) Forty µm brain sections were stained for PV+ interneurons (parvalbumin, red) and cell nuclei (DAPI, blue). Images were prepared using epifluorescence microscopy. Scale bars (500 µm) apply to all panels. N = 3 WT mice.
