# Supplementary material for: Ablation of CCL17‐positive hippocampal neurons induces inflammation‐dependent epilepsy
Source: Epilepsia. 2024 Nov 28;66(2):554–68. doi: 10.1111/epi.18200 (PMC11827734; doi:10.1111/epi.18200)
Supplement: Supplementary file 3 — Figure S2. [file EPI-66-554-s004.pdf]

Figure S2

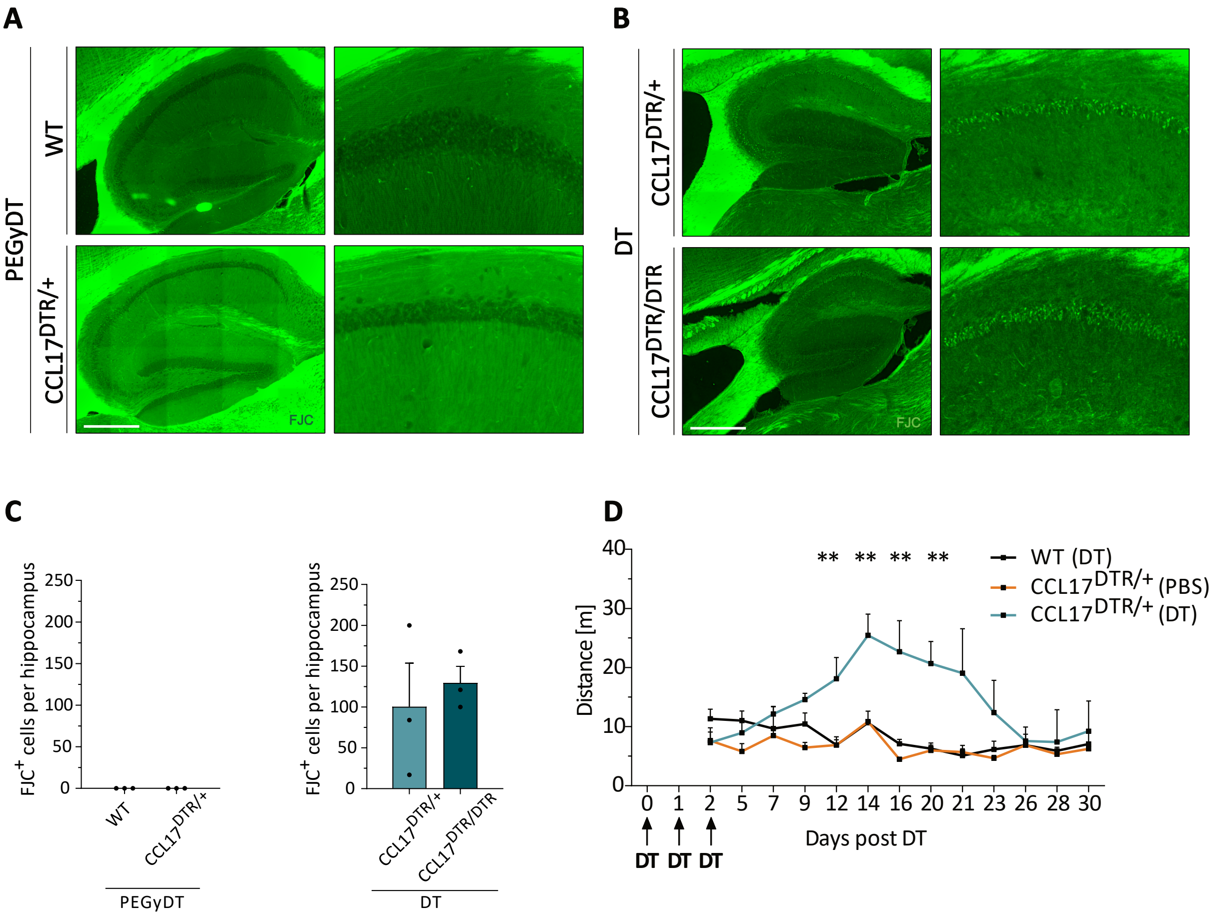

A
B
C
D
PEGyDT
WT
CCL17DTR/+
FJC
DT
CCL17DTR/DTR
FJC+ cells per hippocampus
250
200
150
100
50
0
WT
CCL17DTR/+
PEGyDT
CCL17DTR/+
CCL17DTR/DTR
DT
40
30
20
10
0
Distance [m]
** ** ** **
WT (DT)
CCL17DTR/+ (PBS)
CCL17DTR/+ (DT)
0 1 2 5 7 9 12 14 16 20 21 23 26 28 30
Days post DT
DT DT DT

Figure S2 | Differences in neuronal degeneration and locomotor activity between WT and CCL17^{DTR} mice.

Heterozygous CCL17^{DTR} mice and WT mice were treated at three consecutive days with either 0.4 µg DT or PBS. (A-C) Heterozygous CCL17^{DTR} and WT mice (A) or heterozygous and homozygous CCL17^{DTR} mice (C) were injected with DT or PEGyDT on three consecutive days. Mice were perfused *in situ* and brains were isolated 28 d post-DT. Forty µm brain sections were prepared and stained for DAPI. Degenerating neurons were detected by FJC labeling. Images were prepared using epifluorescence microscopy. Scale bar (500µm) applies to images in the left panels. Representative images are shown. B) Quantification of degenerating neurons in murine hippocampus. Data was tested for statistical significance by ONE-way ANOVA with Bonferroni´s post-hoc test for multiple comparisons (N = 3 CCL17$^{DTR/+}$, 3 CCL17$^{DTR/DTR}$ and 3 WT mice). (D**)** From d2 until d30, locomotion of mice was analyzed. Error bars represent ± SEM. **p < 0.01. WT = wild type; DT = Diphtheria toxin; PBS = phosphate-buffered saline; PEGyDT = pegylated version of DT.
