# Supplementary material for: Ablation of CCL17‐positive hippocampal neurons induces inflammation‐dependent epilepsy
Source: Epilepsia. 2024 Nov 28;66(2):554–68. doi: 10.1111/epi.18200 (PMC11827734; doi:10.1111/epi.18200)
Supplement: Supplementary file 4 — Figure S3. [file EPI-66-554-s002.pdf]

Figure S3

A

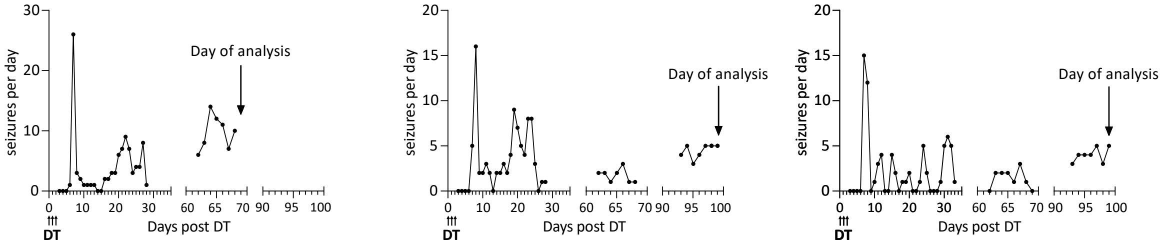

CCL17^{DTR} mouse #1 CCL17^{DTR} mouse #2 CCL17^{DTR} mouse #3

B

CCL17^{DTR} mice

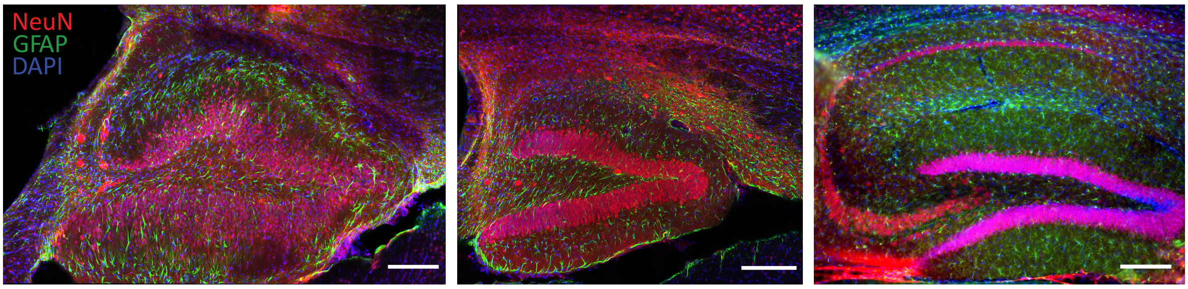

CCL17^{DTR} mouse #1 CCL17^{DTR} mouse #2 CCL17^{DTR} mouse #3

Figure S3 | Long-term analysis of neuroinflammation and seizure activity in CCL17^{DTR} mice

CCL17^{DTR} received 0.4 µg DT i.p. at d0, 1, and 2 post injection. Mice were perfused and brains were isolated 69 or 99 d post-DT. (A) Line graphs depicting the progression of SGS activity for individual CCL17^{DTR} mice for up to 99 days of discontinuous EEG recording. EEG activity was recorded from d1-32, d62-69 and d93-99 post-DT. (B) Forty µm brain sections were prepared and stained for neuronal nuclei marker NeuN (red), for the astrocyte markers GFAP and S100ß (green) and counterstained for cell nuclei with DAPI (blue). Scale bar (500 µm) applies to all panels. Images were prepared using epifluorescence microscopy. N = 3 CCL17$^{DTR/+}$mice.
