# Supplementary material for: Ablation of CCL17‐positive hippocampal neurons induces inflammation‐dependent epilepsy
Source: Epilepsia. 2024 Nov 28;66(2):554–68. doi: 10.1111/epi.18200 (PMC11827734; doi:10.1111/epi.18200)
Supplement: Supplementary file 5 — Figure S4. [file EPI-66-554-s003.pdf]

Figure S4

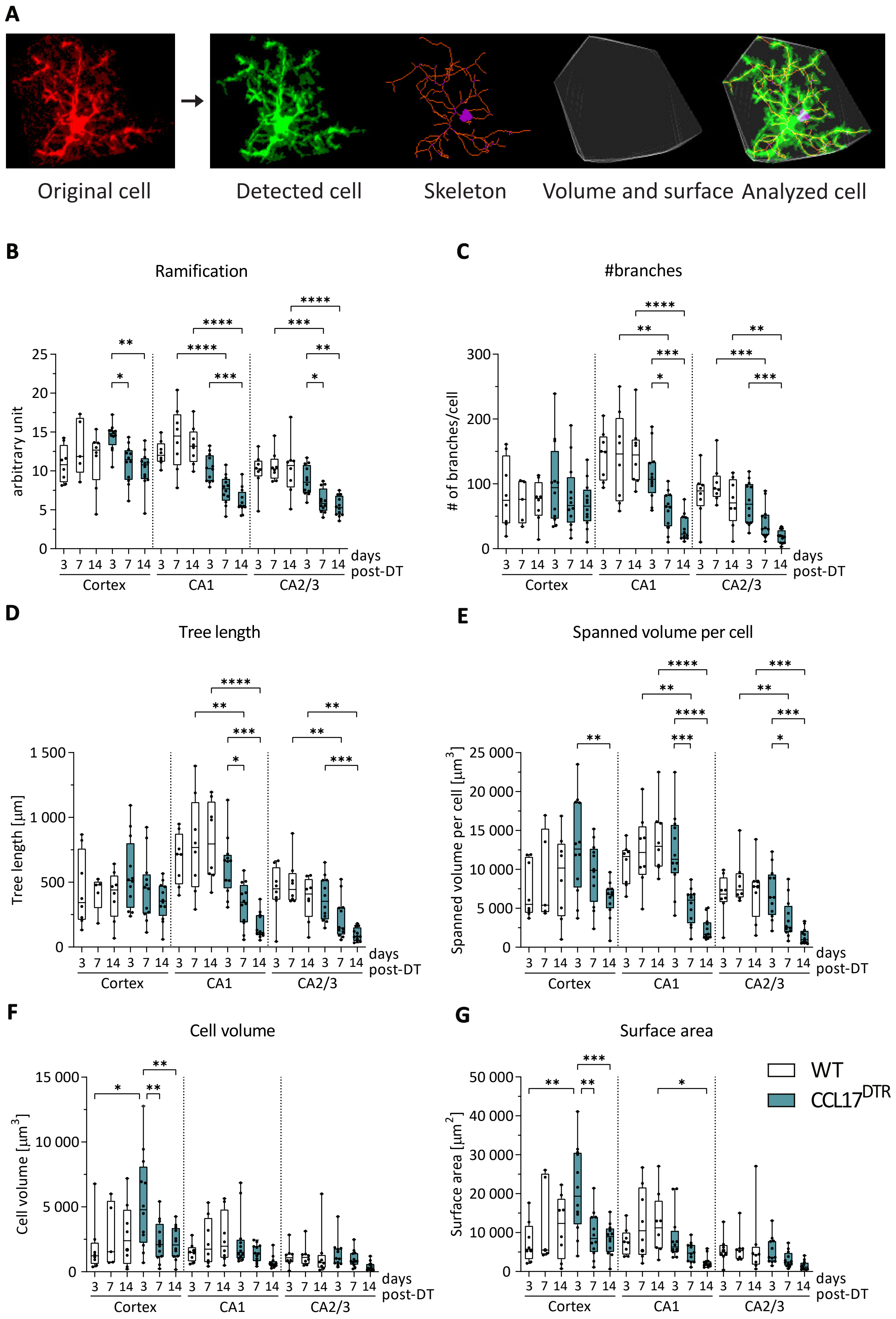
A
Original cell
Detected cell
Skeleton
Volume and surface
Analyzed cell
B
Ramification
arbitrary unit
C
#branches
# of branches/cell
D
Tree length
Tree length [µm]
E
Spanned volume per cell
Spanned volume per cell [µm3]
F
Cell volume
Cell volume [µm3]
G
Surface area
Surface area [µm2]
days post-DT
Cortex
CA1
CA2/3
WT
CCL17DTR

Figure S4 | Ablation of CCL17-expressing cells leads to altered microglia morphology.

Quantification of microglia morphology in murine cortex and hippocampus of $CCL17^{DTR}$ and WT mice that received 0.4 µg DT i.p. at d0, 1, and 2. Mice were perfused and brains were isolated 3, 7 and 14 d post-DT. Forty µm brain sections were prepared and stained for Iba1. Z-stacks were prepared using confocal microscopy and processed in ImageJ (Fiji) using plugins developed by J. Hansen. Four single microglia cells from cortex or hippocampal areas CA1 and CA2/CA3 were analyzed per animal. N = 3 $CCL17^{DTR}$ and 2 WT mice. (A) Overview of the analysis process. (B-G) Ramification, number of branches, tree length of microglia processes, spanned volume per cell, cell volume and cell surface area were analyzed. Data was tested for statistical significance by ONE-way ANOVA with Bonferroni´s post-hoc test for multiple comparisons. ****$p < 0.0001$, ***$p < 0.001$, **$p < 0.01$, *$p < 0.05$.
