# Supplementary material for: Ablation of CCL17‐positive hippocampal neurons induces inflammation‐dependent epilepsy
Source: Epilepsia. 2024 Nov 28;66(2):554–68. doi: 10.1111/epi.18200 (PMC11827734; doi:10.1111/epi.18200)
Supplement: Supplementary file 6 — Figure S5. [file EPI-66-554-s005.pdf]

Figure S5

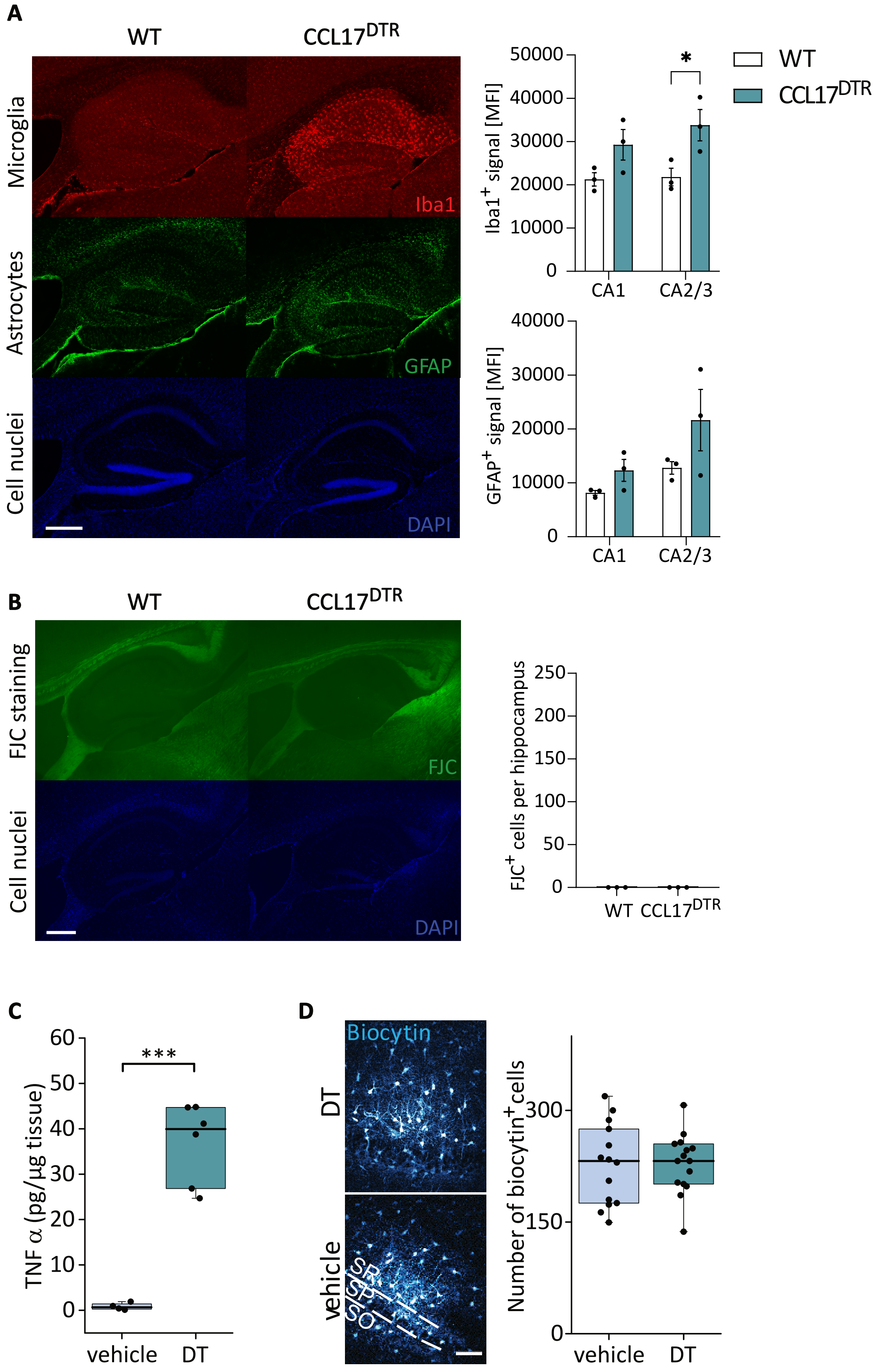
A
WT
CCL17DTR
Microglia
Astrocytes
Cell nuclei
Iba1
GFAP
DAPI
WT
CCL17DTR
*
Iba1+ signal [MFI]
50000
40000
30000
20000
10000
0
CA1
CA2/3
GFAP+ signal [MFI]
40000
30000
20000
10000
0
CA1
CA2/3
B
WT
CCL17DTR
FJC staining
Cell nuclei
FJC
DAPI
FJC+ cells per hippocampus
250
200
150
100
50
0
WT
CCL17DTR
C
TNF α (pg/µg tissue)
60
50
40
30
20
10
0
***
vehicle
DT
D
Biocytin
DT
vehicle
SR
SP
SO
Number of biocytin+cells
300
150
0
vehicle
DT

Figure S5 | Hippocampal TNF concentrations and astrocytic gap junction coupling in CCL17^{DTR} mice 5 d post DT administration.

(A+B) CCL17^{DTR} mice and WT mice received 0.4µg DT i.p. at d0, 1, and 2. Mice were perfused *in situ* and brains were isolated at d5. Forty µm brain sections were prepared and stained for either (A) microglia (Iba1, red), astrocytes (GFAP, green) and cell nuclei (DAPI, blue) or (B) neuronal degeneration (FJC, green) and cell nuclei (DAPI, blue). Images were prepared using epifluorescence microscopy. Scale bar (500µm) applies to upper panels. Representative images are shown. N = 3 CCL17^{DTR} and 3 WT mice. (C) ELISA was performed on hippocampal homogenate to determine the TNF tissue concentration on d5 after the first DT administration. TNF concentrations were significantly increased in the dorsal hippocampus of DT vs. vehicle-injected DTR mice. n = 12 hippocampi from N = 6 mice/group. (D) Representative MIPs depicting biocytin-filled astrocytes labelled with streptavidin-conjugated AlexaFluor® (AF) 647 in hippocampal CA1 str. rad. at d5 post the first DT vs. vehicle injection. Scale bar: 50 µm. No difference in gap junction coupling efficiency between DT and vehicle-treated CCL17^{DTR} mice was found. n = 14-15 cells from N = 3 mice/group. Box plots represent median and quartiles. ***p < 0.001 vs. vehicle. Data were analyzed using either an independent samples t-test or Wilcoxon rank-sum test. CA = *cornu ammonis*; GCL= granule cell layer; str. rad. = *stratum radiatum*; str. pyr. = *stratum pyramidale*.
