# Supplementary material for: Ablation of CCL17‐positive hippocampal neurons induces inflammation‐dependent epilepsy
Source: Epilepsia. 2024 Nov 28;66(2):554–68. doi: 10.1111/epi.18200 (PMC11827734; doi:10.1111/epi.18200)
Supplement: Supplementary file 7 — Figure S6. [file EPI-66-554-s007.pdf]

Figure S6

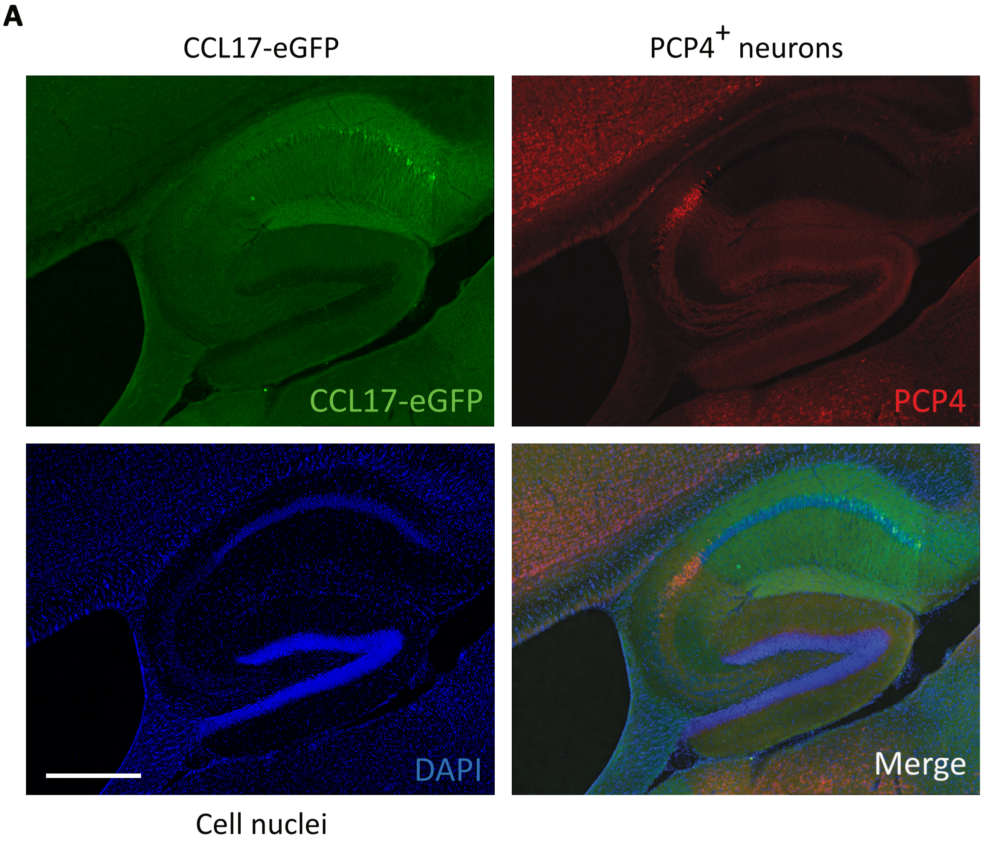
A
CCL17-eGFP
PCP4+ neurons
CCL17-eGFP
PCP4
DAPI
Merge
Cell nuclei

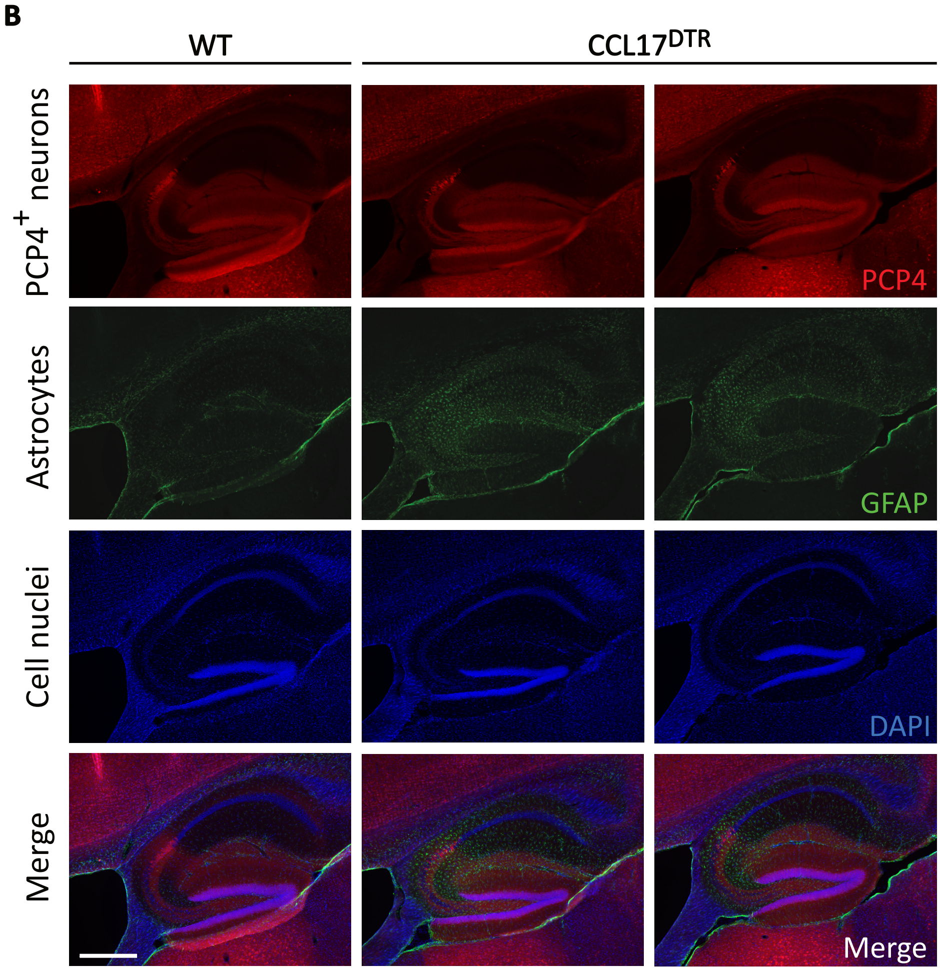
B
WT
CCL17DTR
PCP4+ neurons
Astrocytes
Cell nuclei
Merge
PCP4
GFAP
DAPI
Merge

Figure S6 | Initial neuroinflammation starts in the hippocampal CA2 region.

(A) CCL17-EGFP mice were perfused *in situ* and brains isolated. Forty µm sections were prepared and stained for CA2-located neurons (PCP4, red), CCL17-eGFP (CCL17-eGFP, green) and cell nuclei (DAPI, blue). N = 4 $CCL17^{EGFP}$ mice. (B) $CCL17^{DTR}$ mice and WT mice received 0.4µg DT i.p. at d0, 1, and 2. Mice were perfused *in situ* and brains were isolated at d7. Forty µm brain sections were prepared and stained for neurons located in CA2 (PCP4, red), astrocytes (GFAP, green) and cell nuclei (DAPI, blue). N = 1 WT mouse and 2 $CCL17^{DTR}$ mice. Images were prepared using epifluorescence microscopy. Scale bar (500µm) applies to all panels.
